# Keratinocytes as active regulators of cutaneous and mucosal immunity: a systematic review across inflammatory epithelial disorders

**DOI:** 10.3389/fimmu.2025.1694066

**Published:** 2025-12-17

**Authors:** Felix J. Klimitz, Yizhuo Shen, Federico Repetto, Stav Brown, Leonard Knoedler, Christine J. Ko, Nebal Abu Hussein, William J. Crisler, Taylor Adams, Naftali Kaminski, Christine G. Lian, George F. Murphy, Henry C. Hsia, Bohdan Pomahac, Martin Kauke-Navarro

**Affiliations:** 1 Department of Surgery, Division of Plastic Surgery, Yale School of Medicine, New Haven, CT, United States; 2 Department of Hand, Replantation, and Microsurgery, Burns and Plastic Surgery, BG Trauma Center Berlin, Chair of Hand, Replantation, and Microsurgery, Charité – Universitätsmedizin Berlin, Berlin, Germany; 3 Program in Dermatopathology, Department of Pathology, Brigham and Women’s Hospital, Harvard Medical School, Boston, MA, United States; 4 Departments of Dermatology and Pathology, Yale School of Medicine, New Haven, CT, United States; 5 Section of Pulmonary, Critical Care, and Sleep Medicine, Department of Medicine, Yale School of Medicine, New Haven, CT, United States; 6 Department of Dermatology, Brigham and Women’s Hospital, Harvard Medical School, Boston, MA, United States

**Keywords:** keratinocytes, skin immunity, inflammatory conditions, cytokines, immune pathways, atopic dermatitis, psoriasis, lichen planus

## Abstract

**Background:**

Keratinocytes are increasingly recognized as active regulators of immunity in both skin and mucosal inflammation. Although numerous studies have described their functions in individual conditions, no systematic synthesis has compiled keratinocyte-driven immune mechanisms across the major categories of epithelial injury disorders. We conducted a systematic review to fill this gap and identify both shared and disease-specific pathways that underlie keratinocyte–immune crosstalk in prototypical inflammatory dermatoses.

**Methods:**

A PRISMA-compliant systematic review of studies investigating the role of keratinocytes in immune-mediated skin diseases marked by epithelial injury was performed in MEDLINE, EMBASE, and CENTRAL databases. Included conditions spanned atopic dermatitis (AD), psoriasis, lichen planus (LP), bullous pemphigoid (BP), lupus erythematosus (LE), and graft-versus-host disease (GvHD). These were chosen *a priori* because they are among the most common and clinically relevant inflammatory dermatoses, many with mucosal involvement, and together reflect diverse autoimmune, autoinflammatory, and alloimmune mechanisms. Key outcomes included keratinocyte signaling pathways, immune interactions, and tissue-specific responses.

**Results:**

Eighty-two studies met inclusion criteria: AD (n=49), psoriasis (n=11), LP (n=10), BP (n=3), LE (n=6), and GvHD (n=4). Keratinocytes were consistently implicated in cytokine production (e.g., IL-1β, IL-6, TNF-α, TSLP, IL-33), immune cell recruitment, and antigen presentation (via upregulation of MHC class II and costimulatory molecules such as ICAM-1 or B7). Shared activation pathways included NF-κB, JAK/STAT, and MAPK. Distinct immune profiles emerged across diseases: Th2-skewed responses in AD and BP, Th1/Th17 in psoriasis and LP, and type I interferons in LE and GvHD. Stress keratins (KRT6, KRT16, KRT17) were frequently upregulated and acted as amplifiers of inflammatory signaling. Of the included studies, the majority investigated skin, while mucosal data were largely limited to oral lichen planus and GvHD; mucosal keratinocytes were more often linked to type I interferon–driven apoptosis, whereas cutaneous keratinocytes predominantly amplified inflammation through cytokine and chemokine release, with lupus as an exception.

**Conclusion:**

This systematic review highlights keratinocytes as active regulators rather than passive bystanders in epithelial injury disorders. By integrating diverse inflammatory cues, keratinocytes engage shared and disease-specific pathways that shape immune responses across the spectrum of cutaneous and mucosal inflammation.

## Introduction

The role of keratinocytes, the predominant cell type in the epidermis, extends far beyond forming a physical barrier (1, 2) and contributing to the antimicrobial defense by secreting antimicrobial peptides (AMPs) (3, 4). Emerging research has established keratinocytes as potent immune regulators that initiate and amplify inflammatory cascades in response to various stimuli, thus significantly contributing to the pathogenesis of multiple inflammatory dermatologic diseases (5). Their strategic epidermal position enables them to directly sense external insults, environmental changes, and immunologic triggers, translating these signals into cytokine and chemokine profiles unique to specific dermatologic disorders (6–8). Moreover, keratinocytes establish a dynamic network of communication with various immune cells and act as nonprofessional antigen-presenting cells (APCs) by expressing MHC class I and II molecules, which are essential for antigen presentation to CD8+ and CD4+ T cells, respectively (9–11).

Through cytokine production, antigen presentation, and crosstalk with immune cells, keratinocytes shape both innate and adaptive immune responses in the skin and mucosa (3). Prior reviews have generally approached keratinocyte inflammation in isolated disease contexts, such as atopic dermatitis (AD) and psoriasis (12–14), lichen planus (LP) (15, 16), bullous pemphigoid (BP) (17), lupus erythematosus (18, 19), and graft-versus-host disease (GvHD) (20, 21). However, there are crucial gaps in the comprehensive understanding of commonalities and differences that underlie keratinocyte–immune crosstalk in prototypical inflammatory dermatoses. This systematic review thus synthesizes current knowledge on keratinocyte-mediated immunomodulation by examining four key areas: 1) shared and disease-specific inflammatory pathways activated by keratinocytes, 2) cytokine networks and their effects on immune cell behavior, 3) keratinocyte immune functions across different epithelial tissues, such as skin and mucosa, and 4) translational opportunities and therapeutic targets within keratinocyte-immune communication networks. Taking a multi-disease perspective, this review aims to uncover fundamental principles of epithelial-immune crosstalk and elucidate how local signals shape keratinocyte responses across inflammatory skin conditions.

## Methods

### Search Strategy

This systematic review was conducted according to the Preferred Reporting Items for Systematic Reviews and Meta-Analysis (PRISMA) guidelines (22). The MEDLINE database (PubMed), EMBASE, and the Cochrane Central Register of Controlled Trials (CENTRAL) were queried for relevant articles from inception until November 30^th^, 2024. All studies had to be written in English. Human studies, as well as keratinocyte cell cultures and humanized animal models, were included. Only articles presenting original data were included, while reviews and meta-analyses were excluded. Studies on inflammatory conditions other than AD, systemic and cutaneous LE, LP, pemphigus vulgaris, BP, or GvHD were excluded. These conditions were chosen *a priori* because they represent the most common and clinically relevant inflammatory dermatoses in which keratinocytes are central to pathogenesis, and together they encompass the spectrum of major immunological paradigms (Th2, Th1/Th17, type I IFN, and alloimmune). The references of the included articles were searched for additional eligible studies.

### Registration

This systematic review was registered with the International Prospective Register of Systematic Reviews (PROSPERO) under the registration number CRD42024609092. The authors will make available the review protocol, template data collection forms, data extracted from included studies, and data used for all analyses upon reasonable request.

### Data extraction

The search in Pubmed/MEDLINE, EMBASE, and the Cochrane Central Register of Controlled Trials (CENTRAL) was conducted using the search strings shown in **Supplementary Table S1**. Two reviewers (FK, YS) independently screened all articles by title and abstract. Articles were subsequently analyzed in greater depth through full-text assessment to determine eligibility. Any disagreements regarding the inclusion of individual studies were resolved through consultation with a third author (MK-N). Two authors performed data extraction independently (FK, AS) to ensure accuracy and consistency. Year of publication, country, number of patients, inflammatory condition studied, affected tissue type (skin vs. mucosa), and the specifics of identified keratinocyte function and activation pathways were documented.

### Data synthesis

The data extracted from the included studies were analyzed to identify patterns and group findings related to keratinocyte functions and their immunological interactions in inflammatory skin conditions. Key areas of focus included keratinocyte activation pathways, cytokine release profiles, antigen-presenting capabilities, and interactions with immune cells across different inflammatory contexts. The data were coded and categorized into thematic groups to highlight both shared mechanisms and condition-specific roles of keratinocytes in immune regulation to facilitate a coherent comparison across various skin conditions, providing insights into overarching immune functions and the unique contributions of keratinocytes to disease pathophysiology (**Tables 1**, **2**). Quantitative data were summarized descriptively, and qualitative findings were synthesized to map the diverse roles of keratinocytes in coordinating immunity. Because the included studies were highly heterogeneous and primarily qualitative, formal statistical assessment of publication bias (e.g., funnel plot asymmetry) was not feasible.

**Table 1:** Prominent activation pathways and interaction of keratinocytes with other immune cell types in different inflammatory skin conditions.

Inflammatory skin condition	Activation pathways	Interaction with other immune cells
Atopic Dermatitis	IL-33 and TSLP-driven Th2 inflammation, NF-κB, JAK/STAT, MAPK;	T cells, mast cells, eosinophils, DC
Psoriasis	IL-17 and TNF-α-driven Th17 inflammation NF-κB, JAK/STAT, MAPK;	T cells, DC, neutrophils, macrophages, mast cells
Lichen Planus	IFN-γ and TNF-α-driven Th1 inflammation TLR1/TLR4-mediated NF-κB activation;	T cells, mast cells, DC, pDC, macrophages
Lupus Erythematosus	JAK/STAT activation; CXCL chemokine-driven immune recruitment; Type I IFN (IFN-κ, IFN-α) signaling	T cells, pDC, macrophages, neutrophils
Bullous Pemphigoid	Th2-driven inflammation via IL-4, IL-5, IL-13; TSLP-mediated eosinophil recruitment; IL-16-CD4+ T cell activation	T cells, mast cells, eosinophils, neutrophils, macrophages
Graft-Versus-Host Disease	Acute: Th2/Th17-driven IL-4, IL-5, IL-13, IL-17, and TSLP activation; Chronic: Th1/Th17-mediated IFN-γ, IL-17, and TRAIL receptor signaling	T cells, mast cells, macrophages, DC

IL, Interleukin. TSLP, Thymic Stromal Lymphopoietin. NF-κB, Nuclear Factor kappa-light-chain-enhancer of activated B cells. MAPK, Mitogen-Activated Protein Kinase. EGFR, Epidermal Growth Factor Receptor. PAR2, Protease-Activated Receptor 2. STAT3, Signal Transducer and Activator of Transcription 3. TNF-α, Tumor Necrosis Factor-alpha. IFN, Interferon. JAK/STAT, Janus Kinase/Signal Transducer and Activator of Transcription. TLR, Toll-Like Receptor. CXCL, Chemokine (C-X-C motif) ligand. Th, T-helper cells. DC, dendritic cells. pDC, plasmacytoid dendritic cells.

**Table 2:** Selected examples of stress cytokeratins implicated in inflammatory responses.

Study	Protein	Interacts with	Effect	Context
Cohen et al., 2024 (1)	KRT6 KRT16 KRT17	Proliferation markers: G2M, S-phase genes (MKI67, RPA2, CCNB2)	Correlated at tissue level with hyperproliferation; not cell-autonomous	Psoriasis, CLE, AD, HS
	KRT6A	Differentiation-related genes (EDC locus)	Suggests alternate epithelial differentiation program	Psoriasis, CLE, wound healing
	KRT16	Innate immunity genes (co-regulated, e.g., IFN)	High KRT16/KRT17 ratio associated with enhanced innate immune gene signature	Inflammatory skin disease
	KRT17	High KRT17/KRT16 ratio ➔ upregulation of pathways involved in inflammation: AP-1, ERK/MAPK, TNFα, integrin/β-catenin signaling pathways	Associated with inflammatory transcriptional states	Psoriasis, CLE, AD, HS
Yang et al., 2020 (2)	KRT6A	CXCR4/CD133/EMT regulators	Drives EMT and cancer stem cell transition	Lung adenocarcinoma progression
DePianto et al., 2010 (3) Hobbs et al., 2015 (4) Chung et al., 2015 (5)	KRT17 KRT17	hnRNP K (regulator of gene expression) ➔ forms a transcriptional enhancer Aire (autoimmune regulator, transcriptional regulator) ➔ Aire and KRT17 bind to promotor regions of CXCL19, 11, MMP9, CCL19 NF-kB	Regulates pro-inflammatory chemokine gene expression Enhances transcription of immune response genes and regulates cytokine mRNA levels (e.g. CXCR3 ligands: CXCL9, CXCL10, CXCL11) Promotes Th1/Th17 immune response	Basaloid skin tumors, inflammatory keratinocytes, tumor keratinocytes, promotion of T-cell recruitment, tumor inflammation, inflammatory tumor epithelium Inflammatory skin lesions
Yang et al., 2019 (6)	KRT17	14-3-3σ (adapter/scaffold protein)	Promotes Akt/mTOR signaling, drives proliferation, inflammation, and tumor progression in epithelial tissue	Psoriatic epidermis, tumors
Escobar-Hoyos et al., 2015 (7)	KRT17	STAT3	Enhances STAT3 activation via ubiquitination	Chronic inflammation
Jacob et al., J 2020 (8)	KRT17	Regulates and interacts with nuclear proteins (e.g., LAP2β, Histones, nuclear morphology regulators)	Promotes nuclear shape integrity, histone acetylation, and chromatin accessibility, thereby supporting keratinocyte proliferation through its direct nuclear functions	Cell cultures, tumor keratinocytes, animal models
Nair et al., 2021 (9)	KRT17	γH2A.X, 53BP1, DNA-PKcs, KAP1 (phospho-KAP1), Aire	Supports DNA damage repair and chromatin remodeling	Keratinocytes under genotoxic stress
Liang et al., 2023 (10)	KRT17	YTHDF2 (m6A RNA-binding protein), SKP2/CRL/NEDD8 complex	Promotes degradation of YTHDF2, stabilizes CXCL10 mRNA ➔ increased CXCL10 secretion	CD8+T cell recruitment in colorectal cancer

G2M, S-phase genes, genes active in G2/M and S phases of the cell cycle. MKI6, marker of proliferation Ki-67. RPA2, Replication Protein A2. CCNB2, Cyclin B2. EDC locus, Epidermal Differentiation Complex locus. IFN, Interferon. AP-1, Activator Protein 1. ERK/MAPK, Extracellular signal-regulated kinase/Mitogen-Activated Protein Kinase pathway. TNFα, Tumor Necrosis Factor alpha. EMT, Epithelial–Mesenchymal Transition. hnRNP K, Heterogeneous nuclear ribonucleoprotein K. Aire, Autoimmune regulator. NF-kB, Nuclear Factor kappa-light-chain-enhancer of activated B cells. STAT3, Signal Transducer and Activator of Transcription 3. LAP2β, Lamina-associated polypeptide 2 beta. DNA-PKcs, DNA-dependent protein kinase catalytic subunit. AD, Atopic Dermatitis. CLE, Cutaneous Lupus Erythematosus. HS, Hidradenitis Suppurativa.

## Results

**Figure 1:**
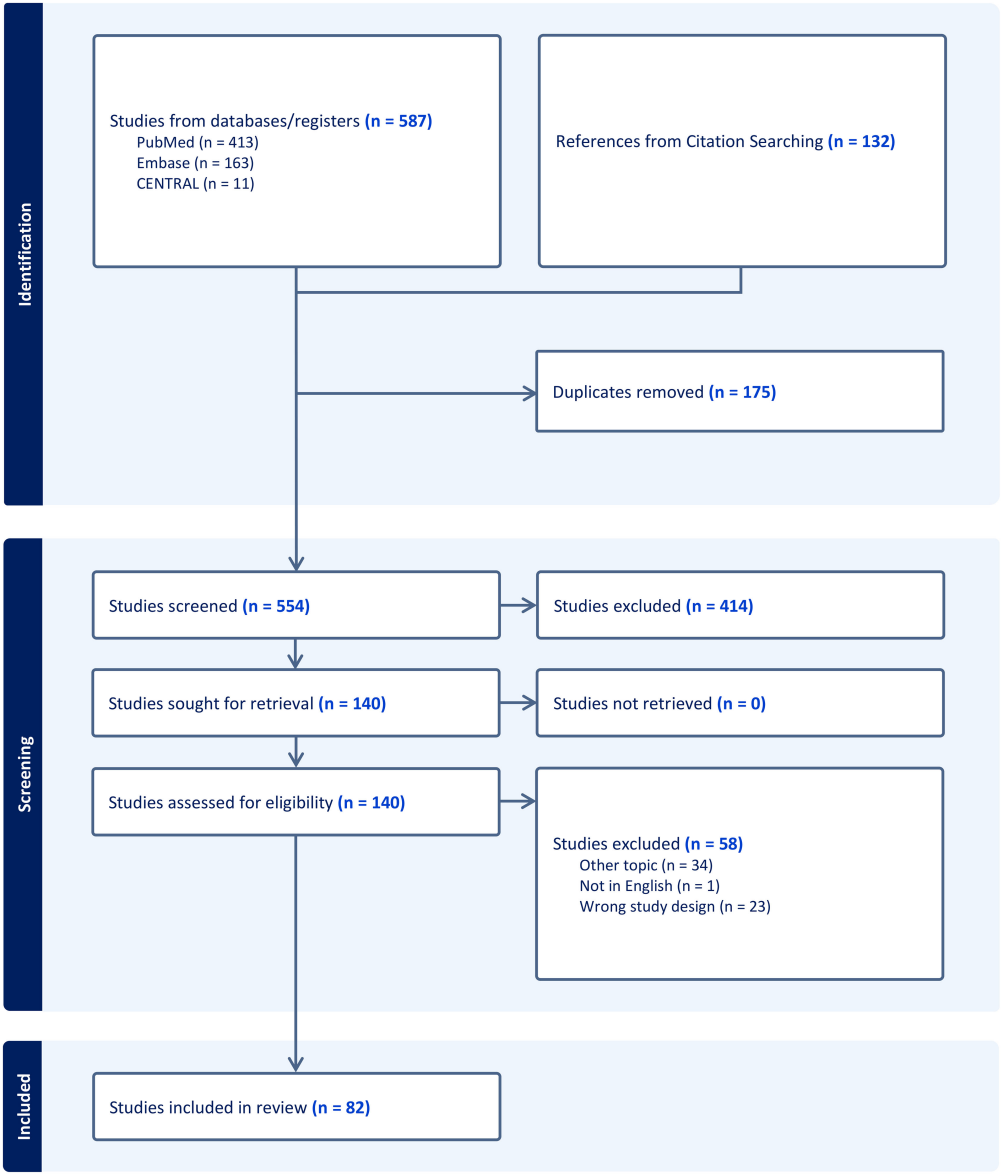
Preferred reporting items for systematic reviews and meta-analyses (PRISMA) flow diagram of study identification, screening, and inclusion.

Our search yielded a total of 587 studies, with 132 additional articles identified through citation searching. Titles and abstracts were screened, leading to the exclusion of 175 additional articles. After full-text analysis, 82 articles were included in the qualitative analysis (23–104). The studies covered a range of inflammatory skin conditions, with AD being the most extensively studied in 49 studies (24–29, 32–36, 40–44, 46–49, 51, 53–55, 58–62, 64, 66–73, 75, 76, 78–80, 82–84, 87, 91, 94). Among those, eleven studies also provided insights into psoriasis (27, 29, 33, 36, 51, 64, 67, 70, 80, 91, 94). LP was addressed in 10 studies (23, 30, 37, 50, 57, 63, 77, 85, 86, 89). GvHD was explored in four studies (31, 45, 52, 81), and lupus erythematosus, including cutaneous (CLE) and systemic lupus erythematosus (SLE), was featured in six studies (38, 56, 57, 65, 74, 88). Lastly, BP was investigated in three studies (39, 56, 90). Lastly, the role of stress cytokeratins in inflammatory conditions was discussed in 10 studies (95–104). A PRISMA flowchart of study identification, screening, and inclusion is presented in **Figure 1**.

The included studies utilized a variety of models to investigate inflammatory skin conditions. Animal models were employed in 23 studies (24, 36, 38, 41, 47, 52, 53, 59, 60, 62, 64, 66, 69, 71, 73, 75, 78, 79, 82, 90, 92, 93). Human keratinocyte cultures were used in 51 studies exploring cellular responses and molecular pathways (23–27, 29, 32–36, 38, 40, 42–44, 46–49, 51, 53–55, 57–60, 62–68, 70–73, 76, 79, 80, 82, 84, 86–89, 93, 94). Additionally, 33 studies incorporated human biopsy samples, offering valuable data on tissue-level changes in human subjects (24, 28, 30, 31, 33, 37–39, 44, 45, 48, 50, 51, 54–57, 61, 63, 65, 73–78, 83, 85, 88, 90–92, 94). The included studies investigated two primary tissue types. Skin was the focus of 64 studies, emphasizing its central role in inflammatory skin conditions (24–29, 31–36, 38–44, 46–49, 51–62, 64–84, 87, 88, 90–94). In contrast, 12 studies examined mucosal tissues (23, 30, 37, 45, 50, 63, 77, 85, 86, 89, 93).

The included studies found various activation pathways across different inflammatory skin conditions and interactions of keratinocytes with different professional immune cell types (see **Table 1**).

### Atopic dermatitis

In AD, keratinocytes play essential roles as initiators and amplifiers of the skin’s inflammatory responses, acting at the interface between genetic predisposition, environmental triggers, microbial colonization, and immune dysregulation. Keratinocytes respond to multiple stimuli, including barrier disruption, allergens, irritants, microbial colonization, and immune dysregulation (62, 67, 75, 91). For instance, *Staphylococcus aureus* influences keratinocyte function through several pathways: bacterial virulence factors and superantigens provoke keratinocytes to produce alarmins such as IL-33 and thymic stromal lymphopoietin (TSLP) via epidermal growth factor receptor (EGFR) signaling pathways independently of traditional Toll-like receptor (TLR) pathways. This promotes type 2 immune responses through recruitment and activation of immune cells like T cells, mast cells, and eosinophils (24). Superantigens further enhance allergen-specific T-cell activation by inducing keratinocyte expression of HLA class II and ICAM-1 via IFN-γ signaling (25). Additionally, keratinocytes express TLR2, TLR4, TLR5, and protease-activated receptor-2 (PAR2), which can be activated by microbial and environmental factors beyond *S. aureus*, driving pruritus and inflammatory cytokine release (26, 32). ATP-mediated purinergic signaling through P2Y2 receptors also triggers IL-33 production, promoting eosinophilic inflammation via EGFR/ERK pathways (35). Further complexity arises as keratinocyte-derived cytokines such as IL-17 activate STAT3, stimulating IL-20 and IL-24 expression, thereby amplifying Th17 and mixed inflammatory responses (40). Keratinocytes can also interact closely with innate lymphoid cells (ILC2s), particularly via IL-33-driven secretion of IL-13, perpetuating type 2 inflammation characteristic of AD (53). Dysregulation of keratinocyte differentiation markers, such as ΔNp63, under chronic IL-13 exposure contributes to persistent inflammation and barrier dysfunction (54). Lastly, keratinocyte-derived IL-24, prominently induced by *S. aureus* and other inflammatory stimuli, activates JAK-STAT3 signaling, exacerbating the production of IL-33 and amplifying type 2 immune responses involving various immune cells (75). Recent single-cell studies have begun to dissect these disease-specific programs at higher resolution. Using scRNA-seq, Zhang et al. identified keratinocytes as major producers of IL-18 in AD but not in plaque psoriasis and demonstrated that keratinocytes in AD express a larger number of severity-associated genes. In contrast, pericytes and vascular endothelial cells showed greater transcriptional alterations among stromal populations in psoriasis (91).

### Psoriasis

Similarly, in psoriasis, keratinocytes play critical roles not only as immune sensors but also as potent amplifiers of inflammation within a multifactorial pathogenic network involving genetic predisposition, immune dysregulation, and environmental triggers. Keratinocyte activation is driven by multiple cytokines, prominently IL-17A, IL-22, TNF alpha, and IFN gamma, which collectively orchestrate the inflammatory environment characteristic of psoriatic lesions (27). Keratinocyte responses are significantly modulated by IL-17A, promoting hyperproliferation, antimicrobial peptide secretion, and chemokine secretion, leading to neutrophil recruitment and lesion perpetuation. In addition, IL-36 and neutrophil extracellular traps (NETs) serve as critical amplifiers of keratinocyte activation in both pustular and plaque psoriasis, further sustaining neutrophilic infiltration and epidermal inflammation (105). Oncostatin M (OSM), signaling through the type II OSM receptor (OSMR), activates STAT3 and MAPK pathways in keratinocytes, enhancing the expression of antimicrobial peptides such as S100A7 and β-defensin 2, and synergizes with IL-17 and TNF-α to exacerbate inflammatory responses (29). Additionally, keratinocyte-derived inflammatory responses are influenced by galectins; galectin-1 (Gal-1) suppresses IL-6 and IL-8 production in IL-17A-stimulated keratinocytes, while Gal-3 similarly regulates responses under IL-4-stimulation (33). The GPR15 ligand (GPR15L) also contributes to keratinocyte-mediated inflammation by stimulating the production of IL-1β, IL-6, and TSLP via MAPK and NF-κB signaling pathways, while simultaneously reducing the expression of barrier-related proteins such as filaggrin and loricrin, exacerbating barrier impairment (36). The JAK/STAT signaling pathway is pivotal in keratinocyte activation, with IFN-γ and IL-6 driving STAT1-dependent transcription and EGFR-mediated chemokine expression, contributing to sustained inflammatory signaling and immune cell recruitment (64, 94). Keratinocytes further facilitate direct interaction with immune cells through IFN-γ-induced upregulation of MHC class II and ICAM-1, engaging with T cells via LFA-1/ICAM-1-dependent costimulatory signaling (70, 80). The hallmark type-3 inflammation seen in psoriasis is largely driven by Th17 and Tc17 cells’ interactions with keratinocytes, driving the robust secretion of chemokines like CXCL8, leading to characteristic neutrophilic infiltrates in psoriatic lesions (91). Keratinocytes also interact dynamically with myeloid cells such as CD11b+ monocytes, macrophages, and dendritic cells, amplifying inflammatory responses through continuous crosstalk with Th1 and Th17 cells (92). Complementing these mechanistic insights, Ma et al. combined single-cell RNA sequencing with spatial transcriptomics to map keratinocyte and fibroblast states in lesional and non-lesional psoriasis skin. Their analyses revealed distinct keratinocyte differentiation trajectories in disease versus health, tightly linked to IL-17A, IL-22, IL-1β, IL-13, and TNF signaling modules. Spatial mapping localized IL36G and S100A7 expression to supraspinous keratinocytes—regions of maximal IL-17A/TNF and IL-36 activity—and highlighted dense fibroblast-keratinocyte crosstalk as a key amplifier of psoriatic inflammation. These data further support keratinocytes as both targets and active participants in cytokine-driven remodeling of psoriatic epidermis (106). These findings support the notion that keratinocytes serve as central inflammatory hubs in AD, whereas vascular and perivascular stromal cells may more strongly amplify inflammation in psoriasis, providing a mechanistic basis for the divergent cytokine milieus summarized above. **Supplementary Table S2** provides an overview of the role of keratinocytes in AD and psoriasis.

### Lichen planus

In LP, keratinocytes play a central role in immune activation and chronic inflammation. LP lesions characteristically reflect a Th1-dominated immune milieu, where keratinocytes actively interface with immune cells through diverse signaling pathways. Activation of TLR1/4–MyD88 signaling initiates IKK-β-mediated NF-κB translocation in keratinocytes, inducing pro-inflammatory chemokines (CXCL1, CXCL10, CXCL11) and intercellular adhesion molecules (ICAM-1) that facilitate lymphocyte infiltration. In addition, cross-activation of JAK2/STAT1 by IFN-γ enhances expression of apoptotic mediators (Fas, TRAIL), coupling innate sensing to cytotoxic injury (23, 37, 89). Keratinocyte activation by IFN-γ and TNF-α signaling axis further amplifies inflammation, through distinct cell death mechanisms, notably necroptosis rather than classical apoptosis. This involves receptor-interacting serine/threonine-protein kinase 3 (RIP3) and phosphorylated mixed lineage kinase domain-like pseudokinase (pMLKL), highlighting a distinct pathological hallmark in LP lesions (57). Concurrently, cytotoxic CD8+ T lymphocytes (CTLs) critically mediate keratinocyte destruction in LP lesions through perforin- and granzyme-dependent pathways, leading to characteristic keratinocyte apoptosis and interface dermatitis (77, 85). Future studies are needed to clarify how TLR-driven and IFN-γ-driven programs intersect to determine necroptosis versus apoptosis in LP. In addition, keratinocytes in LP demonstrate significant antigen-presenting capabilities, with increased expression of MHC class I and II molecules in response to IFN-γ signaling, actively facilitating local T-cell activation and perpetuating chronic inflammation (85). Intracellular signaling pathways, including the PI3K/AKT/mTOR axis, also critically regulate keratinocyte survival, proliferation, and apoptosis. Notably, growth factors such as IGF1 influence these pathways, modulating keratinocyte turnover, lesion stability, and disease chronicity (63, 86). Finally, dysregulated autophagy within keratinocytes, particularly involving autophagy-related protein ATG9B, further shapes the inflammatory landscape in LP. Aberrant autophagy responses in keratinocytes have been shown to modulate T-cell proliferation and apoptosis, reinforcing a chronic inflammatory microenvironment (86). Collectively, these insights position keratinocytes as pivotal players within the multifaceted pathogenesis of LP, directly influencing immune cell dynamics, lesion evolution, and clinical persistence. **Supplementary Table S3** summarizes the role of keratinocytes in LP.

### Bullous pemphigoid

In BP, keratinocytes act as amplifiers of a Th2-dominated autoimmune response directed against the hemidesmosomal protein BP180. Keratinocyte-derived IL-16 facilitates CD4^+^ T-cell recruitment, while IL-4 and IL-13 enhance eosinophilic inflammation via chemokines such as eotaxin and TARC/CCL17 (39). Disruption of BP180 itself triggers keratinocytes to release TSLP via protease-activated receptor-2 (PAR2) and calcium-dependent NFAT signaling, initiating itch through IL-31 and IL-33-mediated sensory neuron activation and type 2 inflammation via mast-cell and eosinophil recruitment (90). Whether PAR2-TSLP signaling originates from basal or suprabasal keratinocytes and how mechanical stress influences this axis remain unclear. These findings highlight that in BP, keratinocytes primarily reinforce antibody-mediated, Th2-driven inflammation and pruritus rather than initiating autoimmunity.

### Lupus erythematosus

In contrast, keratinocytes in LE, particularly cutaneous lupus erythematosus (CLE), are major producers of type I interferons (IFN-κ and IFN-α) that initiate and amplify local inflammation by activating plasmacytoid dendritic cells and promoting chronic autoimmunity (74). Type I IFNs trigger JAK/STAT-dependent chemokine production (CXCL9, CXCL10), driving cytotoxic T-cell recruitment and keratinocyte necroptosis (38). Recent evidence also identifies a distinct role for type III interferons (IFN-λ1–3) in CLE, which, unlike systemic type I IFNs, act locally through epithelial-restricted IFNLR1 signaling. In keratinocytes, IFN-λ engages TLR3-dependent JAK1/TYK2-STAT1/STAT2 pathways, sustaining chronic cytokine and chemokine secretion (CXCL9–11) and persistent epithelial inflammation (88, 107, 108). Importantly, IFN-λ responses are prolonged but less cytotoxic than those driven by type I IFNs, suggesting that chronic IFN-λ activation may sustain subclinical inflammation and barrier injury in photosensitive CLE lesions (107, 109). These insights reveal IFN-λ as a distinct epithelial-restricted interferon axis that could represent a novel therapeutic target complementary to established type I IFN blockade strategies (88, 109). While both BP and LE feature keratinocyte upregulation of MHC class II and local antigen presentation, they differ fundamentally in immune polarization: BP is Th2-driven with eosinophilic infiltration, whereas LE is Th1/type I–III IFN-dominated with cytotoxic T-cell–mediated injury often triggered by UV exposure (**Supplementary Table S4**) (56, 74).

### Graft-versus-host disease

Keratinocytes have emerged as central mediators in the pathogenesis of GvHD, influencing both immune dysregulation and tissue-specific inflammation across acute and chronic manifestations. In acute GvHD, keratinocytes initiate and amplify a predominantly Th2-driven immune response via the production of TSLP, which recruits and activates CD4+ T cells capable of producing IL-4 and IL-22. These cytokines contribute significantly to epidermal inflammation, barrier dysfunction, and the recruitment of additional immune cells, thereby propagating acute tissue injury (31). Chronic lichenoid GvHD, by contrast, is characterized by intense keratinocyte injury mediated predominantly by CD8+ cytotoxic T lymphocytes expressing IFN-γ and IL-17. Cytotoxic T cells induce apoptosis in keratinocytes through perforin and granzyme B–dependent pathways, facilitated by keratinocyte-derived chemokines including CXCR3 and CCR5 ligands, which further enhance T-cell infiltration into the epidermis (31). Additionally, IFN-mediated signaling, particularly involving IFN-γ, enhances keratinocyte apoptosis by upregulating transcription factors such as T-bet in cytotoxic T cells. This upregulation induces production of IFN-inducible proteins (like MxA and CXCL9, CXCL10), reinforcing immune infiltration and inflammation (45). In chronic sclerotic GvHD, keratinocytes participate actively in the Th1-skewed inflammatory milieu characterized by elevated IFN-γ and TNF-α, which can promote keratinocyte apoptosis and contribute to fibrotic remodeling, in part through TGF-β1–mediated signaling. Upregulation of Th1-associated chemokines such as CXCL9–11 and transcriptional activation of STAT1 are well documented in lesional epidermis (110). While engagement of TNF-related apoptosis-inducing ligand (TRAIL) receptors and subsequent caspase-8–dependent apoptosis represent a plausible mechanism based on analogies to other inflammatory skin diseases, direct evidence for TRAIL-R1/R2 involvement in chronic sclerotic GvHD remains limited. Nonetheless, keratinocyte injury and apoptosis are accompanied by the release of alarmins such as HMGB1 and IL-33, which further amplify local Th1 inflammation and tissue sclerosis (31, 111). Additionally, keratinocytes act as direct antigen-presenting cells within GvHD lesions, expressing both MHC class I and II molecules in response to IFN-γ stimulation. In murine K14-OVA models with DC subsets ablated, keratinocytes can prime naïve CD8+ T cells (52). In human skin, keratinocytes present antigen and activate effector or memory T cells (10). However, whether keratinocytes or Langerhans cells serve as the dominant antigen-presenting population remains unresolved, highlighting a key mechanistic gap in cutaneous GvHD. Furthermore, circulating tissue-resident memory T cells (cTRMs) substantially influence chronic GvHD pathology by secreting cytokines including IL-13 and IL-17, directly activating keratinocytes. This results in enhanced local production of TSLP, further amplifying Th2, and perpetuating the inflammatory cycle (81). **Supplementary Table S5** provides an overview of the role of keratinocytes in GvHD.

### The role of cytokeratins in inflammatory skin conditions

Cytokeratins are intermediate filament proteins predominantly expressed in epithelial cells, including keratinocytes, and serve essential structural and immunological functions within the skin. They maintain epithelial integrity, regulate barrier function, and respond dynamically to stress, injury, or inflammation (3). Within inflammatory skin conditions affecting both the skin and mucosa, specific cytokeratins such as keratins 6, 16, and 17 (KRT6, KRT16, KRT17) become markedly upregulated, emerging as critical regulators of innate immunity and inflammation (112). The keratins KRT6, KRT16, and KRT17 are induced as part of the keratinocyte “stress response,” triggered by breaches in the epidermal barrier or inflammatory stimuli. KRT16, for example, directly influences keratinocyte inflammatory signaling through modulation of genes involved in innate immune responses and inflammation, particularly the release of damage-associated molecular patterns (DAMPs), which perpetuate inflammatory cycles in the skin (92). Similarly, KRT17 acts as a key epithelial activation marker prominently expressed during Th1-mediated inflammatory conditions such as psoriasis, but is notably absent in predominantly Th2-driven disorders like AD (94, 113, 114). In psoriasis, KRT17 expression is specifically induced through IFN-γ through the STAT1 signaling pathway. Binding of IFN-γ to its receptor initiates a phosphorylation cascade leading to STAT1 activation, nuclear translocation, and subsequent transcription of the KRT17 gene. Additionally, other cytokines such as IL-6 and leukemia-inhibiting factor (LIF), which also activate STAT1, can induce KRT17 expression. In contrast, cytokines associated with Th2 immunity (e.g., IL-4, IL-10) do not activate this pathway and therefore fail to induce KRT17, reflecting its specific association with Th1-driven inflammation (94). Thus, KRT17 serves as a molecular marker linking keratinocyte stress responses directly to immune polarization in inflammatory skin conditions.

The functional consequence of cytokeratin upregulation in inflammatory conditions extends beyond structural reinforcement. Enhanced expression of these keratins promotes epidermal hyperproliferation, altering keratinocyte differentiation and maturation patterns. Furthermore, cytokeratins directly influence keratinocyte-mediated immune modulation by shaping cytokine and chemokine production, antimicrobial peptide expression, and innate immune cell recruitment, thus actively participating in the perpetuation of chronic inflammation (3, 92, 93). Whether these keratin-immune feedback loops are similarly active in mucosal epithelia or restricted to the skin remains unknown.

## Discussion

The findings of this systematic review highlight the pivotal role of keratinocytes as active and dynamic participants in skin immunity. Beyond their established structural function keratinocytes emerge as key modulators of inflammation, shaping immune responses through cytokine production, antigen presentation, immune cell recruitment, and direct cellular interaction across the spectrum of inflammatory skin conditions analyzed.

### Activation pathways in inflammatory skin conditions

A recurring theme in the pathogenesis of AD in this review is the activation of innate immune signaling pathways within keratinocytes, notably involving NF-κB (32, 36, 47, 82, 84), JAK/STAT (40, 92), and MAPK (36, 78, 94) cascades. These signaling pathways lead to the secretion of pro-inflammatory cytokines such as IL-1β, IL-6, and TNF-α, which collectively orchestrate the inflammatory milieu characteristic of AD. Additionally, keratinocytes significantly contribute to AD pathology through the production of alarmin cytokines such as TSLP and IL-33, which drive type 2 inflammation via recruitment and activation of eosinophils, mast cells, and type 2 innate lymphoid cells (24, 32, 53, 66, 84). Keratinocytes also release IL-25 as a third epithelial alarmin; IL-25 strengthens type 2 immunity and links epithelial injury with itch through PAR2 pathways (44, 47). These findings align closely with studies by Wong et al. demonstrating that IL-31- and IL-33-mediated eosinophil-fibroblast interactions activate the ERK, JNK, and p38 MAPK pathways, enhancing cytokine production, including IL-6 (115). Similarly, Kjellerup et al. confirmed the pivotal role of MAPK-dependent mechanisms within keratinocytes, promoting sustained secretion of IL-1β, IL-6, and TNF-α, thus reinforcing chronic inflammatory responses in AD (116). An important observation arising from our analysis is the critical interplay between keratinocytes and microbial colonization, particularly *S. aureus*. Keratinocytes amplify inflammation in response to *S. aureus* virulence factors, which further enhance barrier dysfunction and cytokine-driven immune cell infiltration, perpetuating disease severity and chronicity (24, 117, 118). These cascades form a feed-forward circuit in which epithelial alarmins (TSLP, IL-25, IL-33) further stimulate STAT5/6 signaling and amplify Th2 polarization. The precise temporal hierarchy and cross-regulation among NF-κB, MAPK, and STAT modules remain poorly defined, representing a critical mechanistic gap for therapeutic targeting.

Our review reveals both shared and distinct keratinocyte activation mechanisms in psoriasis compared to AD. Similar to AD, psoriasis involves keratinocyte-driven activation of the NF-κB, JAK/STAT, and MAPK, which stimulate the production of pro-inflammatory cytokines such as IL-1β, IL-6, and TNF-α (64, 67, 80, 92, 94). However, fundamental differences emerge when examining the dominant immunological axes in each disease. AD is predominantly characterized byTh2 cytokines, such as IL-4 and IL-13, which impair keratinocyte antimicrobial peptide production and promote type 2 inflammation through keratinocyte-derived TSLP and IL-33 secretion (24, 41, 84, 91), mirroring prior reports by Guttman-Yassky et al. (119) In contrast, psoriasis pathology prominently features a T-helper 17 (Th17)-mediated inflammatory axis, driven chiefly by IL-17 and TNF-α, synergistically amplifying keratinocyte activation, antimicrobial defense, and epidermal hyperproliferation (29, 33). This Th17-driven mechanism aligns closely with findings by Sieminska et al., who delineated the TNF-α/IL-23/IL-17 axis as central in psoriasis inflammation (120). Further supporting this, studies by Hawkes et al. and Georgescu et al. characterized psoriasis as a T-cell-mediated inflammatory loop involving IL-23/IL-17 axis, perpetuating keratinocyte activation, proliferation, cytokine secretion, and sustained inflammation (121, 122).

In lichen planus, our findings emphasize a primarily Th1-dominant immune response characterized by robust IFN-γ and TNF-α. These cytokines drive keratinocyte activation and apoptosis primarily through pathways involving JAK2/STAT1 signaling and upregulation of MHC class I, facilitating cytotoxic CD8+ T-cell-mediated damage (50, 57). Additionally, keratinocyte-expressed TLRs, particularly TLR1 and TLR4, amplify inflammatory responses via NF-κB activation, enhancing cytokine secretion and cytotoxic T-cells recruitment through chemokines such as CXCL10 and CXCL11 (6). Our observations are consistent with prior reports, including those by Vičić et al., who confirmed the critical role of the IFN-γ/TNF-α signaling in LP, while also identifying additional contributions from the IL-23/Th17-axis, suggesting a broader immune complexity beyond traditional Th1 classification (123). Shao et al., further provided mechanistic insight, linking IFN-γ-driven MHC class I induction in keratinocytes directly to JAK2/STAT1-mediated susceptibility to CD8+ T-cell cytotoxicity (124). Expanding these insights, Pietschke et al. identified IL-21 as an additional key cytokine sustaining LP inflammation alongside IFN-γ, further delineating LP from purely Th1-driven diseases underscoring its multifaceted immune nature (125).

Our review identified keratinocytes as crucial mediators in bullous pemphigoid, predominantly orchestrating Th2-driven inflammation through secretion of cytokines including IL-16, leading to CD4+ T cells recruitment and amplification of the IL-4/IL-13 axis. Additionally, TSLP signaling from keratinocytes independently drives inflammatory and pruritic pathways separate from classical histamine mechanisms (39, 90). While our findings align with the general consensus in the literature regarding the Th2 cytokine dominance (IL-4, IL-13) in BP (126, 127), notable differences emerge concerning the specific cytokines implicated in disease pathology. Specifically, Liu et al. and Huang et al. emphasize IL-4, IL-5, and IL-13 as primary Th2 cytokines involved in eosinophil recruitment, antibody production, and pruritus (127, 128).

Keratinocyte involvement in systemic lupus erythematosus was found to center on the production of type I IFNs (IFN-κ and IFN-α), driving local inflammation independently of significant immune cell infiltration and enhanced by environmental triggers, particularly UV radiation (74). These observations closely align with findings by Sarkar et al. and Tsoi et al., highlighting keratinocyte-derived IFN-κ and IFN-α as key modulators of local inflammation and photosensitivity (129). Our assertion regarding inflammation independent of immune cell infiltration potentially supplements other studies that have emphasized substantial infiltration by plasmacytoid dendritic cells and T cells as a hallmark of SLE pathogenesis (129–132). Keratinocyte-derived IFNs might initially drive inflammation, which is later perpetuated by plasmacytoid dendritic and T-cell infiltration. In CLE, type I IFN-driven JAK/STAT signaling was strongly implicated, promoting chemokine (CXCL9, CXCL10) production and facilitating keratinocyte necroptosis via an autocrine IFN amplification loop (38). Our findings concur with studies by Robinson et al., who confirmed that type I IFNs prominently activate JAK/STAT signaling pathways in CLE, perpetuating chronic inflammation through sustained chemokine secretion (133). Conversely, our identification of type III IFN (IFN-λ) signaling via TLR3 as central to CLE inflammation (88), is less supported by current literature, which predominantly emphasizes type I IFNs in CLE pathogenesis (129, 130, 134, 135). This suggests that while IFN-λ signaling pathways may represent emerging areas of research interest, they currently remain secondary to type I IFN-driven mechanisms widely recognized in CLE pathogenesis. However, it remains unsolved how keratinocytes balance cytoprotective DNA-repair signaling with cGAS-STING activation, and why mucosal keratinocytes show reduced sensitivity to UV-induced IFN-κ release.

Our findings underscore that GvHD exhibits distinct keratinocyte activation pathways across its clinical variants, with notable differences between acute and chronic forms. Acute GvHD involves multiple T-helper subsets, notably Th1, Th2, and Th17 responses. Brüggen et al. described acute GvHD as being predominantly Th2-driven, characterized by keratinocyte-derived TSLP and cytokines such as IL-4, IL-5, and IL-13, recruiting and activating T cells (31). Additionally, Strobl et al. further nuanced this model, reporting circulating tissue-resident memory T cells (cTRMs) as crucial mediators in GvHD, enhancing keratinocyte-driven inflammation through secretion of IL-13, IL-17, and TSLP, thereby suggesting an integrated Th2/Th17 mechanism (81). However, these Th2-centric observations contrast with prior studies emphasizing Th1 and Th17 dominance in acute GvHD. Boieri et al. demonstrated, in a rat model, robust Th1 gene expression signatures associated with rapid-onset acute disease (136). Supporting this, Nikolic et al. showed a critical role for Th1 cell in initiating acute GvHD, while associating Th2 responses primarily with organ-specific damage, notably within the skin (137). Overall, aGvHD appears to be a multifaceted immunological condition, in which Th1 and Th17 responses appear more critical for initial disease pathogenesis. Chronic GvHD presents further complexity, exhibiting distinct immunopathological patterns in its lichenoid and sclerotic forms, including upregulation of IFN-inducible genes and damage-response pathways, but the relative contributions of Th1, Th17, and profibrotic mechanisms differ, resulting in their distinct clinical and histopathological presentations. Our review highlights chronic lichenoid GvHD as primarily Th1/Th17-mediated, characterized by IFN-γ and IL-17-driven keratinocyte apoptosis through granzyme and perforin released by infiltrating CD8+ T cells (31, 45). These findings align closely with Wenzel et al., who emphasized the critical role of CXCR3 ligands (CXCL9, CXCL10) in mediating lymphocytic infiltration and sustaining inflammation in chronic lichenoid GvHD (138).

The importance of type I IFN-driven apoptosis via T-bet-expressing cytotoxic T cells, as indicated by chemokine-mediated (CXCL9) infiltration, further underscores the involvement of IFN pathways in chronic forms of GvHD (31, 45). Additionally, we identified evidence suggesting that keratinocytes might directly present antigens to naïve CD8+ T cells, potentially independent of Langerhans cells (LCs) and dermal dendritic cells (dDCs) (52). However, earlier research by Kubota et al. strongly positions LCs and dDCs as indispensable initiators of cutaneous immune responses in GvHD, highlighting the ongoing complexity regarding cellular antigen-presenting roles within skin immunity (139).

Collectively, our systematic review highlights shared and distinct keratinocyte activation pathways across inflammatory skin conditions that span a wide range of prototypic diseases, inclusive of eczematous (AD), psoriasiform (psoriasis), interface vacuolar (GvHD), interface lichenoid (LP), and autoimmune bullous (BP). Commonly activated pathways, including NF-κB, JAK/STAT, and MAPK, consistently mediate production of inflammatory cytokines such as IL-1β, IL-6, and TNF-α. Despite these overlapping signaling cascades, each condition manifests distinct immunological signatures driven by specialized cytokine profiles and cellular interactions.

- Atopic dermatitis is primarily Th2-driven, characterized by IL-4, IL-13, TSLP, and IL-33, promoting eosinophil and mast cell infiltration.
- Psoriasis exhibits a clear Th17 dominance, with IL-17 and TNF-α synergizing to amplify keratinocyte hyperproliferation and antimicrobial peptide expression.
- Lichen planus is Th1-skewed, driven by IFN-γ and TNF-α, although recent evidence also highlights significant IL-23/Th17 involvement, reflecting a broader inflammatory spectrum.
- Bullous pemphigoid displays a clear Th2-mediated inflammatory response, characterized by IL-4, IL-5, and IL-13, eosinophil recruitment and pruritus.
- SLE and CLE are distinguished by type I IFN signaling, particularly IFN-κ and IFN-α, strongly linked to photosensitivity, chemokine-mediated inflammation, and chronic disease pathology.
- Graft-versus-host disease illustrates differential activation depending on clinical subtype: acute GvHD involves integrated Th1, Th17, and Th2 responses, whereas chronic lichenoid GvHD demonstrates dominant Th1/Th17 involvement, notably mediated by IFN-γ and cytotoxic CD8+ T cells.

### Mechanistic integration and knowledge gaps

Across these conditions, keratinocyte activation converges on NF-κB, JAK/STAT, and MAPK cascades that determine whether cells adopt inflammatory, apoptotic, or regenerative fates. Emerging single-cell and spatial-omics data suggest that pathway dominance may depend on keratinocyte state and microenvironmental cues, including microbial metabolites, mechanical stress, and local cytokine gradients. However, the hierarchical organization and temporal dynamics of these pathways remain poorly defined. Dissecting these circuits will be crucial to identifying intervention points capable of restoring immune balance without impairing barrier repair. A recent cross-disease fibroblast atlas integrating more than 350,000 fibroblasts from 32 single-cell datasets across 23 skin conditions and multiple tissues exemplifies the power of harmonized transcriptomic analyses to delineate shared and disease-specific stromal states (140). A comparable, unified keratinocyte atlas does not yet exist but would be highly valuable for systematically comparing keratinocyte differentiation programs and cytokine responses across AD, psoriasis, lupus, bullous pemphigoid, and graft-versus-host disease.

### Comparison of immunological roles of keratinocytes in skin and mucosa

Our review highlights that keratinocytes play essential but distinct immunological roles in skin versus mucosal tissues across multiple inflammatory conditions, shaped by tissue-specific differences in gene expression, immune cell populations, environmental exposures, and microbial colonization.

In atopic dermatitis, mucosal keratinocytes similarly engage Th2-mediated responses, yet typically exhibit reduced inflammation severity and accelerated healing, reflecting intrinsic mucosal tissue differences in barrier integrity and immunological response mechanisms (4, 141–143). In psoriasis, while mucosal involvement is uncommon, affected mucosal keratinocytes share the Th17-driven pathology but generally demonstrate less pronounced hyperproliferation, underscoring distinct regulatory mechanism present in mucosal epithelia (3, 144–146). For LP, keratinocyte activation in the skin is predominantly mediated by Th1-type responses, characterized by IFN-γ and TNF-α signaling that induces keratinocyte apoptosis and inflammatory cell infiltration. Mucosal LP also involves Th1 responses but may differ significantly in cytokine profiles due to the unique immune cell populations and local microbiome influences, reflecting differential immune activation in mucosal versus cutaneous environments (143, 147, 148). In BP, skin keratinocytes actively mediate Th2-dominant inflammation, secreting cytokines including IL-4, IL-5, and IL-13, facilitating eosinophil recruitment and blister formation. Mucosal BP similarly exhibits Th2-driven immunopathology but may differ clinically and histologically due to the intrinsic immunological milieu and specific environmental conditions of mucosal surfaces (147). In lupus erythematosus, IFNs, particularly IFN-κ and IFN-α, amplify inflammatory cascades in the skin characterized by cytokines such as IL-6, IL-1β, and TNF-α, and chemokines (CXCL9, CXCL10), resulting in sustained immune cell infiltration. UV exposure further exacerbates this process by inducing keratinocyte apoptosis and nuclear antigen release, intensifying autoimmune responses (130, 131, 134, 149, 150). By contrast, mucosal keratinocyte involvement in SLE is less clearly delineated, reflecting differences in immune cell populations, antigenic exposures, and environmental stimuli, notably reduced UV exposure. Mucosal keratinocytes likely contribute via type I IFN-mediated pathways but with distinct cytokine environments and potentially milder inflammatory responses relative to skin (129, 130, 134, 151). In GvHD, chronic sclerotic GvHD primarily involves Th1 responses with keratinocyte-mediated fibrosis driven by TGF-β signaling and TRAIL receptor activation (31, 111, 152, 153). In contrast, mucosal GvHD involves keratinocyte-mediated inflammation through distinct type I IFN–dependent pathways, recruitment of T-bet+ cytotoxic T cells, and epithelial apoptosis. Additionally, mucosal environments, display unique immunoregulatory interactions, such as Langerhans cell-mediated suppression of cytotoxic T cells, thus differing significantly from skin-associated immune dynamics (45, 139). Recent evidence from facial vascularized composite allotransplantation further underscores this distinction: while skin rejection is dominated by T cell–mediated cytotoxicity, mucosal rejection shows a distinct enrichment of CD19^+^ B cells, plasma cells, and B-1–like populations that colocalize with areas of keratinocyte injury. This mucosa-specific B-cell signature highlights that keratinocyte-driven immune responses in mucosa are supported by fundamentally different cellular partners than in skin, providing mechanistic insight into why mucosal inflammation often appears earlier and more severe than cutaneous inflammation in comparable settings (154).

In summary, keratinocytes universally function as central modulators of inflammation across skin and mucosal surfaces but exhibit differences shaped by tissue-specific environmental, genetic, and immunological factors. Skin keratinocytes typically engage robust inflammatory responses characterized by pronounced barrier dysfunction, hyperproliferation, and intense cytokine-driven immune cell infiltration. Conversely, mucosal keratinocytes commonly demonstrate moderated inflammation, more rapid healing responses, and distinct cytokine and chemokine expression profiles influenced by their unique immune environment. Understanding these tissue-specific differences in keratinocyte immunobiology has critical implications for therapeutic strategies targeting inflammatory conditions of skin and mucosal epithelia.

### The role of cytokeratins in inflammatory skin conditions

Cytokeratins (CKs) constitute a critical subgroup of intermediate filament proteins (Types I and II) that are essential for forming the cytoskeletal architecture of epithelial cells, including keratinocytes. The functional cytoskeletal network generally arises from the co-expression of paired type I and type II keratin genes, conferring mechanical stability and structural integrity to epithelial tissues (155). The expression patterns of cytokeratins in surface epithelia, notably in skin and mucosa, exhibit greater complexity compared to internal epithelia (e.g., gastrointestinal lining) (156). Consequently, cytokeratin profiles have long served as valuable biomarkers for epithelial cell identity, differentiation, stress responses, and immune activation states, significantly enhancing our understanding of disease mechanisms and progression in various pathological conditions. Emerging evidence has demonstrated the role of CKs beyond structural functions, identifying their significant involvement in modulating inflammatory and immune responses within inflammatory skin disorders (157). For example, in atopic dermatitis, keratins such as Keratin 1 (KRT1) and Keratin 10 (KRT10) critically contribute to maintaining epidermal barrier integrity and participate in complex inflammatory networks through cytokine interactions, notably with IL-18, linking epidermal differentiation processes directly to inflammatory responses characteristic of AD (4, 158, 159).

In contrast, keratin expression in psoriasis highlights distinct stress-responsive keratins—including Keratins 6A-C (KRT6A-C), Keratin 16 (KRT16), and Keratin 17 (KRT17)—which serve as molecular markers indicating keratinocyte activation and cellular stress responses (95). These keratins function prominently as epithelial-derived alarmins, activating innate immune responses, altering keratinocyte adhesion, promoting cell migration, and sustaining inflammatory signaling cascades (14, 112, 160). Notably, stress-induced keratins KRT6A-C, KRT16, and KRT17 regulate pathways involving AP-1, TNF- α, MAPK, and NF-kB signaling, thereby directly influencing immune and inflammatory gene expression within the epidermis (95, 112). Interestingly, KRT6, KRT16, and KRT17 are typically not expressed in healthy epidermal tissues, and their expression is usually restricted to specialized epithelial compartments such as skin appendages (e.g., hair follicles, sebaceous and sweat gland, nail plates) and palmoplantar regions (161). Their induction within inflamed epidermis thus provides crucial diagnostic and mechanistic insights, identifying keratinocytes actively engaged in pathological inflammatory and hyperproliferative processes. Thus, keratin expression profiles not only reflect the structural state of epithelial tissues but also actively mediate inflammatory cascades, linking cellular stress responses with immune modulation in inflammatory skin conditions. Ichikawa et al. demonstrated that KRT6, KRT16, and KRT17 are not only markers of hyperproliferation but also occur in non-hyperproliferative, atrophic epidermis characterized by hydropic degeneration and dermal inflammation, as seen in discoid lupus erythematosus and lichen planus. This suggests that these keratins likely reflect a reparative or wound-healing response to basal-layer damage, potentially regulated by cytokines secreted by infiltrating inflammatory cells (162).

**Figure 2:**
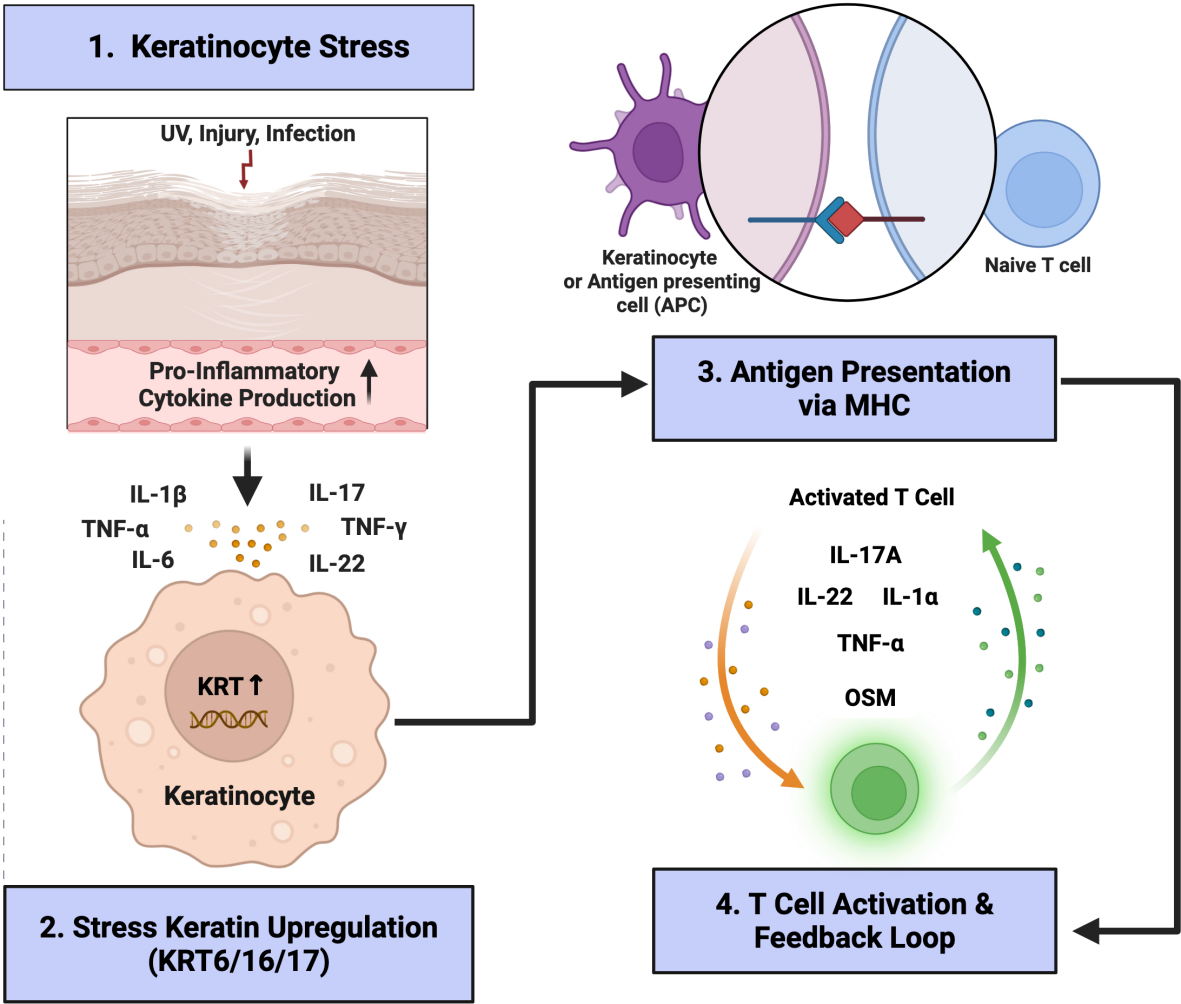
Overview of keratinocyte stress and immune feedback loop in inflammatory skin disease. Schematic representation of the shared keratinocyte-immune interaction cycle underlying chronic inflammatory dermatoses. Environmental stressors such as ultraviolet irradiation, mechanical injury, or infection induce keratinocyte stress and the release of pro-inflammatory mediators including IL-1β, IL-6, TNF-α, IL-17, and IL-22 (Step 1). In response, keratinocytes up-regulate stress keratins (KRT6, KRT16, and KRT17) (Step 2), which are hallmarks of epithelial activation. These activated keratinocytes can function as non-professional antigen-presenting cells, expressing MHC molecules and co-stimulatory ligands that promote T-cell priming and activation (Step 3). Activated T cells subsequently secrete cytokines such as IL-17A, IL-22, IL-1α, TNF-α, and oncostatin M (OSM), thereby amplifying the inflammatory circuit and sustaining epidermal remodeling (feedback loop, Step 4). Depending on the prevailing cytokine milieu, this loop may culminate in keratinocyte hyperproliferation (e.g., psoriasis) or apoptosis and interface injury (e.g., lupus erythematosus, lichen planus, graft-versus-host disease). The figure summarizes the unifying pathway through which stressed keratinocytes perpetuate inflammation and adaptive immune activation across distinct inflammatory skin disorders.

Among stress-induced cytokeratins, KRT17 stands out due to its pronounced immunomodulatory properties, influencing inflammatory pathways, immune cell recruitment, and cytokine networks across multiple skin conditions. In psoriasis, KRT17 expression is strongly upregulated in suprabasal keratinocytes, correlating closely with epidermal hyperproliferation and inflammation. Unlike differentiation keratins (KRT1, KRT10), which typically maintain epidermal integrity in healthy skin, KRT17 does not replace these markers but is instead co-expressed, signifying an alternative differentiation pathway characteristic of chronic inflammation (95). Crucially, KRT17 expression is directly driven by pro-inflammatory cytokines including IFN-γ, IL-17, IL-22, and TGF-β1 through activation of STAT1, STAT3, and ERK1/2 signaling pathways. The cytokine-mediated induction of KRT17 creates a pathogenic K17-T cell-cytokine positive-feedback loop amplifying both keratinocyte proliferation and inflammation (94, 100, 113, 114, 163). Furthermore, peptides derived from KRT17 can function as autoantigens, eliciting T-cell responses that further perpetuate inflammation, thereby establishing a chronic autoimmune loop. This cytokine–KRT17–immune cell axis plays a critical role in sustaining inflammation in psoriasis (100). These immune-modulatory functions of KRT17 extend beyond psoriasis. Luo et al. demonstrated in allergic contact dermatitis that KRT17 enhances T-cell recruitment through the upregulation of the chemokine CCL20, exacerbating local inflammatory responses. Moreover, KRT17’s regulatory role has been highlighted in tumor immunology, particularly colorectal cancer, where KRT17 interacts directly with the m6A reader YTHDF2, stabilizing CXCL10 mRNA and enhancing CD8+ T-cell recruitment to the tumor microenvironment, thus potentiating anti-tumor immune responses and efficacy of immune checkpoint blockade therapies (104, 164). Similarly, in cutaneous tumors, KRT17 interacts with RNA-binding proteins like heterogeneous nuclear ribonucleoprotein K (hnRNP K), regulating the expression of pro-inflammatory chemokines such as CXCL9, CXCL10, and CXCL11, thereby linking keratinocyte stress responses to chronic inflammatory signaling pathways (99). The stress-induced upregulation of KRT17, which promotes antigen presentation and establishes a pro-inflammatory feedback loop via T cell activation and cytokine amplification is shown in **Figure 2**.

Interestingly, although KRT17 expression strongly correlates with increased tissue-wide proliferation, studies suggest that its role at the single-cell level does not directly induce proliferation. Instead, KRT17 predominantly functions as an immunological modulator, emphasizing its contribution to keratinocyte stress response pathways rather than cellular division itself (95). In addition, KRT17 has emerged in other disease contexts as well, such as in idiopathic pulmonary fibrosis, where a distinct population of aberrant basaloid cells—likely derived from alveolar type II cells—ectopically express KRT17, indicating a pathological epithelial reprogramming (165). Given its multifaceted immune-regulatory role, KRT17 and other stress keratins emerge as a therapeutic target in chronic inflammatory skin diseases such as psoriasis, atopic dermatitis, cutaneous lupus erythematosus (CLE), and hidradenitis suppurativa (HS). For example, therapeutic approaches targeting KRT17 expression using antisense oligonucleotides or small interfering RNA (siRNA) demonstrate promise in reducing inflammation and disease severity (100, 112, 113). **Table 2** provides an overview of selected stress keratins implicated in inflammatory responses (95–104).

**Figure 3:**
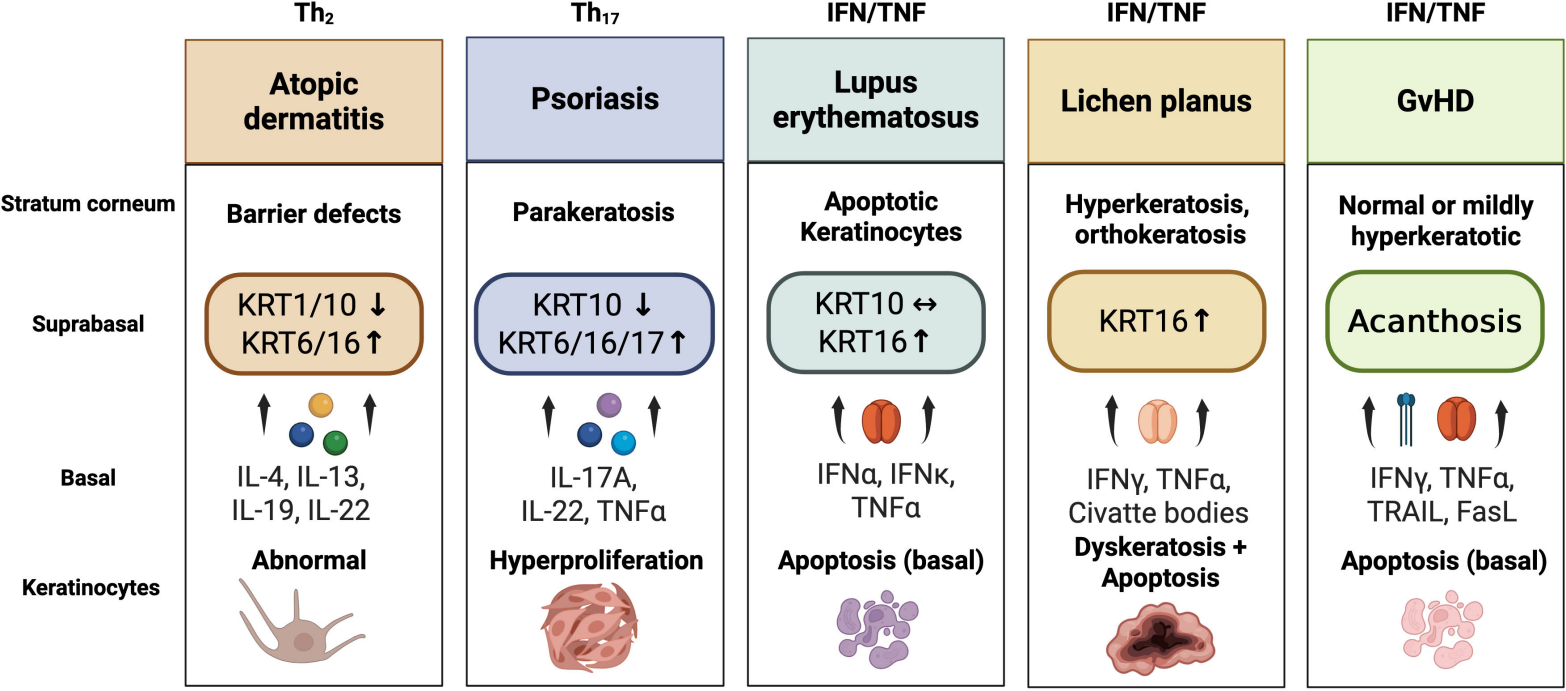
Disease-specific keratinocyte activation patterns and cytokeratin expression across inflammatory dermatoses. Distinct cytokine environments shape characteristic keratinocyte responses and cytokeratin expression profiles across inflammatory skin diseases. In atopic dermatitis (Th2-driven), cytokines such as IL-4, IL-13, IL-19, and IL-22 suppress differentiation keratins (KRT1/10) while inducing stress keratins (KRT6/16) in the suprabasal layers, leading to impaired barrier function. In psoriasis (Th17-dominant), IL-17A, IL-22, and TNF-α stimulate suprabasal upregulation of KRT6/16/17, accompanied by loss of KRT10, resulting in parakeratosis and marked keratinocyte hyperproliferation. In lupus erythematosus (IFN/TNF-mediated), type I interferons (IFN-α, IFN-κ) together with TNF-α induce basal keratinocyte apoptosis and mild KRT16 elevation, whereas KRT10 expression remains unchanged. In lichen planus (IFN-γ/TNF-α-driven), cytotoxic cytokines provoke basal keratinocyte apoptosis and Civatte-body formation, with compensatory KRT16 induction in suprabasal layers; histologically, this manifests as hyperkeratosis and dyskeratosis. In chronic cutaneous graft-versus-host disease (IFN-γ/TNF-α/TRAIL/FasL-mediated), cytokine-induced caspase-8 activation causes basal apoptosis and vacuolar degeneration, followed by secondary acanthosis, but no consistent keratin signature. Bullous pemphigoid, in contrast, represents an autoantibody-mediated subepidermal blistering disease (anti-BP180/BP230) without defined keratinocyte or cytokeratin alterations. Collectively, these disease-specific patterns illustrate how cytokine milieu and epidermal compartmentalization determine distinct keratinocyte activation states across inflammatory dermatoses.

In summary, cytokeratins critically contribute to inflammatory skin conditions, serving both structural and immunological roles (**Figure 3**). In AD, KRT1 and KRT10 maintain epidermal integrity and modulate cytokine-driven inflammation. In conditions such as psoriasis and lichen planus, stress-induced keratins (KRT6, KRT16, and KRT17) are highly upregulated, contributing to keratinocyte hyperproliferation, migration, and acting as epithelial alarmins that amplify immune signaling. Among these, KRT17 notably modulates immune responses through the regulation of cytokines (IFN-γ, IL-17, IL-22) and chemokines (CXCL9, CXCL10, CXCL11, CCL20).

### Emerging cytokeratin-targeted therapies

There is an emerging therapeutic interest in stress-induced cytokeratins such as KRT6, KRT16, and KRT17. Though direct inhibitors are not yet in clinical use, RNA interference strategies (e.g., antisense oligonucleotides or siRNA) targeting KRT6 and KRT16 are under preclinical investigation for their potential to attenuate keratinocyte activation and downstream immune signaling (166–168). In addition, indirect suppression through cytokine blockade (e.g., IL-17A inhibitors such as secukinumab and ixekizumab) or JAK/STAT inhibition (e.g., tofacitinib) has demonstrated the capacity to downregulate KRT6 and KRT16 expression by interfering with their transcriptional inducers (121, 169). Topical retinoids like tazarotene also contribute to the normalization of keratinocyte differentiation and reduce KRT16 levels (112). Among cytokeratins, KRT17 presents the most promising therapeutic target due to its unique role in amplifying inflammation via a pathogenic cytokine–KRT17–T cell feedback loop. Strategies to directly suppress KRT17 expression using antisense oligonucleotides or siRNA have shown encouraging results in reducing keratinocyte-driven inflammation in preclinical psoriasis models (167, 168, 170). These approaches aim to disrupt key pro-inflammatory signaling pathways, including STAT1/3 and ERK1/2, and diminish chemokine-driven immune cell recruitment (167, 169). Furthermore, the potential for targeting KRT17 in immuno-oncology and chronic inflammatory conditions underscores its broad clinical relevance (100, 171). Collectively, these evolving strategies highlight the therapeutic promise of modulating cytokeratin expression to treat chronic inflammatory skin diseases.

## Conclusion

This systematic review highlights the pivotal role of keratinocytes as active coordinators of immune responses in inflammatory skin conditions, functioning beyond mere structural barriers to become central initiators and amplifiers of inflammation. Across conditions such as atopic dermatitis, psoriasis, lichen planus, bullous pemphigoid, lupus erythematosus, and GvHD, keratinocytes consistently exhibited critical involvement in cytokine-driven inflammation, immune cell recruitment, and local tissue responses. Common signaling pathways, specifically NF-κB, JAK/STAT, and MAPK emerged as central mediators of keratinocyte-driven inflammatory mechanisms. While Th2 cytokines such as IL-4, IL-13, IL-33, and TSLP dominated the inflammatory landscape in AD and bullous pemphigoid, Th1 and Th17 immune responses, primarily mediated by IFN-γ and IL-17, characterized psoriasis, lichen planus, and specific forms of GvHD. Notably, keratinocytes demonstrated dynamic interactions with diverse immune cell populations, including T cells, mast cells, eosinophils, and dendritic cells, underscoring their immunomodulatory capacity. Importantly, keratinocyte behavior differed between skin and mucosal tissues. Mucosal keratinocytes typically exhibited less severe inflammatory responses, faster resolution of inflammation, and reduced barrier dysfunction, suggesting inherent tissue-specific differences in immunological responses. Additionally, the role of cytokeratins, particularly stress-associated keratins such as KRT6, KRT16, and notably KRT17, emerged as critical in modulating inflammatory processes, indicating promising therapeutic targets for future interventions. Overall, these findings underscore the integral role of keratinocytes in driving and sustaining inflammatory processes in dermatological disorders. However, major knowledge gaps remain. Few studies directly compare these diseases within unified experimental frameworks, limiting understanding of shared versus distinct keratinocyte signaling programs. Moreover, most existing data are derived from skin-only models, with little insight into mucosal counterparts or early pre-inflammatory stages. Future research should aim to further clarify the molecular mechanisms underlying these interactions, ultimately guiding the development of novel targeted therapies. Specifically, future studies should focus on (1) integrating single-cell and spatial transcriptomic approaches to delineate keratinocyte–immune cell interactions at high resolution; (2) comparing cutaneous and mucosal keratinocyte responses to identify tissue-specific immunoregulatory mechanisms; (3) investigating how environmental factors such as the microbiome, UV exposure, and mechanical stress modulate keratinocyte activation; and (4) translating these insights into targeted interventions that modulate keratinocyte signaling (e.g., JAK/STAT or KRT17 pathways) for disease-specific therapy. Elucidating these aspects will not only enhance mechanistic understanding but also facilitate precision medicine approaches for inflammatory dermatoses.

## Limitations

Despite the comprehensive nature of this systematic review, several limitations must be acknowledged. First, inherent biases in the included studies, such as variations in study design, sample size, and experimental models, may influence the generalizability of our findings. The inclusion of both human and animal studies, as well as *in vitro* keratinocyte models, introduces heterogeneity in the data, which could affect the consistency of reported immune mechanisms. Additionally, the review is limited by the availability of published literature, potentially overlooking unpublished or non-English studies that may provide further insights into keratinocyte function in inflammatory skin conditions. Another limitation arises from the focus on selected inflammatory conditions, meaning that other relevant dermatological diseases with immune involvement were not assessed. Furthermore, the reliance on studies with varying methodologies may impact the comparability of cytokine expression patterns and immune cell interactions across conditions. Another limitation is the potential for publication bias. Most included studies were laboratory-based or small observational reports that may preferentially publish positive or mechanistically supportive findings, while negative or null results are likely underrepresented. This bias could lead to an overestimation of the consistency of keratinocyte-driven immune mechanisms across conditions. Future systematic reviews incorporating unpublished data or registered preclinical studies could help mitigate this effect. A further limitation is that we did not perform a formal integration or meta-analysis of single-cell and spatial transcriptomic datasets across the included dermatoses. Instead, individual scRNA-seq studies were referenced to illustrate disease-specific keratinocyte programs. Consequently, this review cannot provide a unified high-resolution atlas of keratinocyte states comparable to the recently published fibroblast atlas in inflammatory skin disease, and important subtleties in keratinocyte heterogeneity across disorders may remain unresolved. Future efforts aggregating and harmonizing existing single-cell datasets will be essential to construct such a keratinocyte atlas and delineate shared versus disease-specific keratinocyte states.
