# Supplementary material for: Keratinocytes as active regulators of cutaneous and mucosal immunity: a systematic review across inflammatory epithelial disorders
Source: Front Immunol. 2025 Dec 17;16:1694066. doi: 10.3389/fimmu.2025.1694066 (PMC12753988; doi:10.3389/fimmu.2025.1694066)
Supplement: Supplementary file 1 [file DataSheet1.zip › Supplementary Table 1.DOCX]

**Supplementary Content**

**Table S1.** Search strings in PubMed/MEDLINE, EMBASE and the Cochrane Central Register of Controlled Trials (CENTRAL)

| **PubMed/MEDLINE** | **EMBASE** | **CENTRAL** |
| --- | --- | --- |
| (keratinocytes[MeSH Terms] OR keratinocytes [Title/ Abstract]) AND (immune [MeSH Terms] OR immunity [MeSH Terms] OR immune response[Title/Abstract] OR immunity [Title/Abstract]) AND (("Systemic Lupus Erythematosus" [Title/Abstract] OR "Cutaneous Lupus" [Title/Abstract] OR lupus erythematosus[MeSH Terms]) OR "Lichen Planus"[MeSH Terms] OR lichen ruber [Title/Abstract] OR pemphigus [MeSH Terms] OR bullous pemphigoid[Title/Abstract] OR atopic dermatitis [Title/Abstract] OR "Graft vs Host Disease"[MeSH Terms]) | (keratinocyte.ti,ab.) AND (("immune response" OR immunity OR "immune activity").ti,ab.) AND ((systemic lupus erythematosus OR "cutaneous lupus".ti,ab. OR "lupus erythematosus".ti,ab.) OR lichen planus/ OR "lichen ruber".ti,ab. OR pemphigus vulgaris/ OR "bullous pemphigoid".ti,ab. OR "atopic dermatitis".ti,ab. OR graft versus host disease/) | (keratinocytes AND ("immune response" OR immunity OR immune)) AND ("Systemic Lupus Erythematosus" OR "Cutaneous Lupus" OR "lupus erythematosus" OR "Lichen Planus" OR "lichen ruber" OR pemphigus OR "bullous pemphigoid" OR "atopic dermatitis" OR "Graft vs Host Disease") |
