# Supplementary material for: Keratinocytes as active regulators of cutaneous and mucosal immunity: a systematic review across inflammatory epithelial disorders
Source: Front Immunol. 2025 Dec 17;16:1694066. doi: 10.3389/fimmu.2025.1694066 (PMC12753988; doi:10.3389/fimmu.2025.1694066)
Supplement: Supplementary file 1 [file DataSheet1.zip › Supplementary Table 2.DOCX]

**Table S2 Summary of Studies Investigating the Role of Keratinocytes in Atopic Dermatitis and Psoriasis**

| **Author & Year** | **Country** | **Model** | **Tissue** | **Trigger** | **Key Pathways in Keratinocytes** | **Keratinocyte Response** | **Interaction with Immune Cells** |
| --- | --- | --- | --- | --- | --- | --- | --- |
| **Atopic Dermatitis** | | | | | | | |
| Al Kindi et al., 2020 (1) | United Kingdom, Japan | Human keratinocyte cultures, skin biopsies, mouse model | Skin | Second immunoglobulin-binding protein (Sbi) from S. aureus | **EGFR** | IL-33 and TSLP release | **T cells:** Recruited for Type 2 immunity  **Mast cells** and **eosinophils:** inflammatory amplification |
| Ardern-Jones et al., 2007 (2) | United Kingdom | Human keratinocyte cultures and peripheral blood mononuclear cells | Skin | **Staphylococcal superantigen B** (SEB) | **IFN-γ** signaling | **HLA class II** and **ICAM-1** ↑ → antigen presentation | **SEB-reactive** and **allergen-specific CD4+ T cell** activation → **Th2** cytokine (IL-4) ↑ → inflammation |
| Aries et al., 2016 (3) | France | Human keratinocyte cultures | Skin | Poly I:C, TNFα, IL-4, and IL-13, stratum corneum tryptic enzyme, Th1, Th2, and Th17 cytokines | **TLR5**  **Protease-activated receptor-2 (PAR2)** | Antimicrobial peptide (AMP) production, cytokines (e.g., **TSLP, IL-18, IL-8**) and chemokines (**MCP-3, MDC, MIP-3α**) ↑, differentiation marker **involucrin** ↓ | **CD4+ T cells:** activates **Protease-activated receptor-2** with Th1, Th2, and Th17  **Langerhans cells** recruiment |
| Bilsborough et al., 2006 (4) | USA | Human skin biopsies and peripheral blood | Skin | **IL-31** by T cells | IL-31 receptor A (**IL-31RA**) on keratinocytes and macrophages | Chemokine production (**TARC/CCL17, MDC/CCL22**) | **Th2 cells**: Produce IL-31, IL-4, and IL-13 to enhance inflammation.  **Macrophages**: Express IL-31RA. Skin-homing memory **T cells** (**Cutaneous lymphocyte antigen-positive)**: produce IL-31→inflammation and pruritus in AD lesions. |
| Buddenkotte et al., 2005 (5) | Germany | Human keratinocyte cultures | Skin | Serine proteases (e.g., trypsin, mast cell tryptase) | **PAR2** and **NF-κB** | Serine proteases → **PAR2**↑→ **ICAM-1**↑.  **PAR2**↑ in AD lesions. | **T cells** recruitment via ICAM-1  **Mast cells** release tryptase → PAR2 activation in keratinocytes |
| Dai et al., 2011 (6) | Japan | Human keratinocyte cultures | Skin | House dust mite (HDM) allergens | **NLRP3** inflammasome | Release of **IL-1β** and **IL-18** | Recruiment/activation of immune cells, inflammation, **Th1** and **Th2** responses |
| Dai et al., 2020 (7) | Japan | Human keratinocyte cultures | Skin | House dust mite (HDM) allergens | Purinergic P2Y2 receptors (**ATP/P2 Receptor), EGFR/ERK, Intracellular Calcium Mobilization** | **IL-33** production | IL-33 activates **Type 2 Innate Lymphoid Cells, Th2 Lymphocytes, Mast Cells, Basophils** → Th2 cytokines and eosinophilic inflammation |
| Haertlé et al., 2023 (8) | Germany | Human keratinocyte cultures and peripheral blood mononuclear cells (PBMCs) | Skin | IL-17 | **JAK-STAT** (IL-20R1/IL-20R2 and IL-22R1/IL-20R2 heterodimer complexes) | Inflammatory mediators IL-20, IL-24, CCL20 and IL-36γ↑ | **Th17 cells** secrete IL-17.  **Other T cells** (Th2/Th1) amplify inflammation.  **Myeloid cells and mast cells** respond to IL-20 and IL-24 secretd by keratinocyte. |
| He et al., 2008 (9) | USA | Mouse model | Skin | -- | -- | **TSLP** | TSLP enhances Th2 cytokine production (IL-4, IL-13) by skin-infiltrating **CD4+ T** cells.  TSLP recruits **eosinophils** to the dermis → allergic inflammation. TSLP polarizes **DCs** to favor Th2 differentiation, partly through OX40L expression. |
| Howell, et al., 2006 (10) | USA, Germany, and Italy | Human keratinocyte cultures | Skin | **Th2 cytokines**, IL-4 and IL-13 | STAT-6, **inhibiting NF-κB** and the **TNF-α signaling pathway** | **HBD-3** (human beta-defensin-3) ↓ | **Peripheral blood mononuclear cells** (PBMC), **T-helper cells** (especially Th2), **natural killer cells** |
| Howell et al., 2008 (11) | USA | Human keratinocyte cultures and skin biopsies | Skin | **Th2 cytokines IL-4** and **IL-13** | -- | S100 calcium-binding protein A11 (S100/A11) and p21↓→ keratinocyte differentiation ↓, filaggrin (FLG) ↓ and HBD-3↓ → weakened skin barrier and antimicrobial defense | **T cells** are attracted by the cytokine milieu → Th2 inflammation.  **Mast cells** amplify cytokine signaling |
| Hvid et al., 2011 (12) | Denmark | Human keratinocyte cultures and skin biopsies | Skin | **IL-25** | IL-17Rh1 receptor | FLG synthesis ↓ → skin barrier dysfunction | **DCs/Mast cells:** produce IL-25  **Th2 cells** promoted by IL-25 |
| Ishibashi et al., 2006 (13) | Japan | Human keratinocyte cultures | Skin | **IL-5, IL-10, IL-13** by M. globosa  **IL-4** by M. restricta | -- | GM-CSF and CTACK (cutaneous T cell-attracting chemokine) secretion ↑ | Th2 cytokines (IL-5, IL-4) recruits and activates **eosinophils**  CTACK attracts **Memory T cells**  GM-CSF increases antigen presentation in  **Langerhans cells** and **DCs** |
| Jang et al., 2017 (14) | South Korea | Human keratinocytes cultures and mouse model | Skin | **House Dust Mite** | **TLR1, TLR6, TLR9** and nucleotide-binding oligomerization domain 2 (NOD2)  **NF-κB** | **pro-Th2 cytokines** (IL-25 and IL-33) ↑ through TLR1/6 signaling | IL-25 and IL-33 promote Th2-type immune responses via **ILC2** activation.  Keratinocyte-derived cytokines recruits **eosinophils, mast cells, and Th2 cells** → allergic inflammation. |
| Jin et al., 2014 (15) | South Korea, China | Human keratinocyte cultures and skin biopsies | Skin | Th17 Cytokines (IL-17A, IL-17F, IL-22)  House Dust Mites (HDM) | S100A8 and S100A9 → TLR4 and RAGE receptors | S100A8 and S100A9 expression↑  **IL-33 expression**↑ | IL-33 amplifies **Th2** cytokine production, promoting inflammation.  S100A8/A9-mediated signaling recruits **Monocytes and Neutrophils**  Th2 cytokines activates **eosinophils** |
| Junghans et al., 1996 (16) | Germany | Human keratinocyte cultures | Skin | IL-4 by **Th2 cells** | **IL-4 receptor** | B7/BB1 expression↑ | **T-cells:** B7/BB1-mediated costimulation and SEB-induced activation. |
| Kim et al., 2020 (17) | South Korea | Human keratinocytes cultures and mouse model | Skin | **IL-17A** from group 3 innate lymphoid cells (ILC3s) | -- | IL-33 production↑  Epidermal hyperplasia | **ILC2s:** Release IL-5 and IL-13, promoting type 2 inflammation.  **Neutrophils:** Major source of IL-1β, stimulating IL-17A secretion by ILC3s.  **Eosinophils and mast cells:** Recruited to the skin via IL-17A-driven pathways, contributing to inflammation. |
| Kubo et al., 2021 (18) | Japan | Human keratinocyte cultures and skin biopsies | Skin | **Type 2 cytokines** IL-13 and IL-4 | -- | IL-13 inhibits **ΔNp63** downregulation during differentiation → inflammatory cytokines (e.g., IL-1β, IL-33) ↑ | IL-13 and IL-33 stimulates **Th2 cells**  IL-33 activates **ILC2s**, releasing IL-13 |
| Kumagai et al., 2017 (19) | Japan | Human keratinocyte cultures and skin biopsies | Skin | PAMPs such as double-stranded RNA | **TLR3** | Overexpression of **ΔNp73** → **NF-κB** activation → **TSLP**↑ | TSLP activates **ILC2s** and induces **Th2** differenetiation |
| Lee et al., 2016 (20) | South Korea | Human keratinocyte cultures | Skin | TSLP | **JAK2/STAT3 (**STAT3 phosphorylation) | AMPs (S100A7 and human β-defensin 2 (hBD2)) ↓ | TSLP promotes type 2 immune responses, **recruiting Th2 cells** and other inflammatory immune cells indirectly by modulating keratinocyte-derived cytokines. |
| Lou et al., 2017 (21) | USA, China | Human keratinocyte cultures and transgenic mouse model | Skin | **IL-22** | **IL-22 receptor** (IL-22R)  Gastrin-releasing peptide **(GRP)/GRPR pathway** | Th2 cytokine (TSLP and IL-33) ↑, pruritus, and Th2 inflammation | **CD4+** and **CD8+ T cells** (involved in Th2 and Th22 responses)  IL-22 recriuts **eosinophils, mast cells, and macrophages** |
| Lu et al., 2022 (22) | China | Human keratinocyte cultures and mouse model | Skin | IL-20 | IL-20R1 and IL-20R2 | Calcium influx, inflammatory cytokine transcription, and AD-related gene expression ↑ | **IL-20** signals to sensory neurons in adjacent sensory ganglia → neuro-immune communication, itch and inflammation  **Keratinocyte-derived cytokines** indirectly activates **T cells** and **DCs** |
| Lugović et al., 2005 (23) | Croatia | Skin biopsies and peripheral blood analysis | Skin | High **IFN-γ** production by activated **Th1** in acute AD lesions | IFN-γ receptor | Inflammation | **Th1** cells producing IFN-γ dominate in acute AD lesions  **Langerhans Cells (LCs)** present antigens to Th cells; increased in AD lesions |
| Luo et al., 2024 (24) | Taiwan | Mouse and human keratinocyte models; epicutaneous S. aureus infection in delipidized skin, and keratinocyte cultures. | Skin | S. aureus | TLR2, RIPK3 and MLKL | Necroptosis and IL-33 | IL-33 activates **ILC2s** → IL-13 release and dermal inflammation  **Neutrophils and macrophages** are recruited to inflamed skin |
| Meng et al., 2019 (25) | Ireland, Germany, Qatar, USA | Human keratinocyte cultures and mouse dorsal root ganglion neurons | Skin | Poly(I:C) | **TLR3** | upregulation and release of proinflammatory cytokines  **TSLP, ET-1** (Endothelin-1), and **TNF-α** | TSLP and ET-1 activates **sensory neurons** → itch and inflammation.  TSLP modulates **DCs** and enhances **Th 2** immune responses |
| Moriwaki et al., 2019 (26) | Japan | Human keratinocyte cultures | Skin | **S. aureus** CpG DNA | **TLR9** | IL-1α secretion | Recruiment and activation of immune cells → inflammation. |
| Murthy et al., 2012 (27) | Canada | Mouse model | Skin | Disintegrin and metalloprotease 17 (ADAM17) deficiency, environmental stress (allergen, trauma) | **Notch, AP-1** | **Notch**↓/AP-1 activity ↑ → TSLP, IL-33 and G-CSF ↑ | TSLP → Th2 cytokine (IL-4, IL-15) ↑  IL-17 ↑ by **Th17 cells** and **γδ T cells**.  **IL33** → Dermal **mast cell** infiltration and IgE elevation.  MHC II expression by **APCs**↑.  G-CSF → expansion of **Gr-1+CD11b+ myeloid cells** → myeloproliferative disease. |
| Ogawa et al., 2020 (28) | Japan | Human keratinocytes cultures and mouse model | Skin | Oxidative stress and electrophilic stimuli | **NF-E2-related factor 2** (NRF2) activation | **IL-1α** and **TSLP** production  Small proline-rich protein 2 (**SPRR2**) and detoxifying enzymes (e.g., NQO1, glutamate-cysteine ligase) ↑ | Keratinocyte-derived inflammatory mediators recruits **CD4+ T cells, mast cells,** and **eosinophils**  NRF2-driven signals condition **DCs** for T cell memory generation |
| Park et al., 2021 (29) | South Korea | Human keratinocyte cultures | Skin | -- | **NLRP3 inflammasome activation** (including ASC and caspase-1) | **IL-1β** and **IL-18 production** via canonical inflammasome pathway | **Th1, Th2, Th17,** and **Th22 cytokine pathways**  **Mast cells, macrophages, neutrophils** |
| Proper et al., 2024 (30) | United States | Human keratinocyte cultures and skin biopsies | Skin | IL-13 | **Aryl hydrocarbon receptor (AHR) signaling pathway**, **STAT6** | **Normal:** AHR attenuate IL-13-induced gene expression, such as CCL26 (eotaxin-3)  **AD:** AHR dysregulated | Altered keratinocyte gene expression → recruit and activate innate immune cells (**eosinophils,** potentially **macrophages** and **DCs)** |
| Qian et al., 2024 (31) | China | Human keratinocyte cultures and mouse model | Skin | IL-24 produced by keratinocytes in response to MRSA infection | **JAK-STAT3** | IL-33 production | **ILC2s** produce IL-4 and IL-13 in response to IL-33 → **Type 2 Immunity**, inflammation  **Macrophages and Neutrophils** are recruited to the site for defense and inflammation. |
| Salimi et al., 2016 (32) | United Kingdom | Human keratinocyte cultures and skin biopsies | Skin | Inflammation | **--** | B7-H6 ↑ | NKp30 receptor activated by B7-H6 in ILC2→ **NF-κB** activation →**Type 2 Immunity** (IL-4, IL-5, IL-13) by ILC2s  **Myeloid-Derived Cells** also express B7-H6 → inflammatory microenvironment |
| Savinko et al., 2012 (33) | Finland | Human keratinocyte cultures and skin biopsies | Skin | **TNF-α, IFN-γ,** amplified by environmental triggers (e.g., allergens, irritants, SEB) | **IL-33–ST2 signaling pathway, NF-κB** and **MAPK** | IL-33 ↑ (also produced by **macrophages** and **endothelial cells)** | **IL-33** interacts with ST2 receptors on immune cells (**mast cells, eosinophils, Th2)** → Th2 cytokines ↑ |
| Sawada et al., 2019 (34) | Japan | Human keratinocyte cultures and skin biopsies | Skin | **PGE2** | **PAR2** internalization via EP2 receptors | TSLP ↓ | TSLP recruits **Th2 cells**, activates **basophils** and **ILC2s** → Type 2 inflammation. |
| Su et al., 2020 (35) | China | Human keratinocyte cultures and skin biopsies | Skin | **IL-4** and allergens | **--** | Transglutaminase 3 (**TGase3**) expression ↑  TGase3 binds DC-SIGN, a receptor on monocyte-derived dendritic cells (MoDCs) | DC-SIGN binding activates **NF-κB** in **MoDCs** → **IL-6** production and **Th1 polarization** |
| Suárez-Fariñas et al., 2013 (36) | USA | Human skin biopsies | Skin | Intrinsic AD: **Th17** (IL-17, IL-23, CCL20).  Extrinsic AD: **IgE**, Th2 cytokines.  Both: **Th2** (IL-4, IL-13), **Th22** (IL-22), and **Th1** (IFN-γ) **cytokines** | -- | -- | **T cells:** Elevated CD3+, CD8+, and Th2, Th17, and Th22 subsets infiltrate lesions.  **DCs:** Myeloid dendritic cells (CD11c+), **Langerhans cells** (CD1a+), and **plasmacytoid dendritic cells** (BDCA2+ in extrinsic AD).  **Eosinophils:** Predominantly recruited in extrinsic AD.  **Neutrophils:** Increased in intrinsic AD due to Th17 activity. |
| Takai et al., 2014 (37) | Japan | Human keratinocyte cultures | Skin | **TLR3, TLR5,** and **TLR2-TLR6 ligands** (e.g., double-stranded RNA, flagellin, diacylated lipopeptides) | **NF-κB Activation** | TSLP production | TSLP-activated **DCs** secrete chemokines → recruit and polarize **Th2 Cells** to produce IL-4, IL-5, and IL-13 → inflammation and epithelial barrier disruption  **Innate Immune Cells** contributes to chronic inflammation |
| Yoshikawa et al., 2013 (38) | Japan | Human keratinocyte cultures | Skin | **IFN-γ** | -- | **IFN-γ** → **IL-32, IL-1B, CXCL1**, and **IL-8** ↑ from AD donors compared to non-AD donors  **IL-32** → **pro-IL-1β** ↑→ **IL-1β** ↑ by inflammasome, potentially involving **NLRP2** | **Th1 cells** secretes IFN-γ → keratinocyte activation.  **Monocytes** and **Langerhans cells** may also respond to IL-32 via inflammasome to cytokine and chemokine cascades (IL-8, CXCL1)→immune recruitment and activation. |
|  |  |  |  |  | **Atopic Dermatitis (AD) & Psoriasis (PS)** |  |  |
| Boniface et al., 2007 (39) | France | Human keratinocyte cultures | Skin | **OSM** | AD: -- | Migration, hyperplasia, and pro-inflammatory gene expression | **AD: Th2 cells** produce OSM and cytokines (**IL-4** and **IL-13** ) → inflammation↑ and antimicrobial peptide production ↓ |
|  |  |  |  |  | PS: Type II OSM receptor (OSMR), **STAT3**/**MAP kinase** | Antimicrobial peptides (**S100A7, β-defensin 2**). production ↑ | **PS: Th17** and **Th1** secrete IL-17, TNF-α, OSM → keratinocytes produce chemoattractants like IL-8 and CXCL5 →neutrophil infiltration |
| Corrêa et al., 2022 (40) | Brazil | Human keratinocyte cultures and skin biopsies | Skin | **IL-4** and **IL-17A; Galectin-1** (Gal-1) and **Galectin-3** (Gal-3) | IL-4 → Th2 inflammation (AD);  IL-17A → Th17 inflammation (PS) | Gal-1, Gal-3 → migration;  **Gal-1** → **IL-6, IL-8**, RANTES ↓ (PS)  **Gal-3** → IL-6 ↓ (AD) | Th2 cytokines in AD → **eosinophils** and **mast cells**.  Th17 cytokines in PS → **T cells, neutrophils, and macrophages**. |
| Dainichi et al., 2022 (41) | Japan | Human keratinocyte cultures and mouse model | Skin | **GPR15L** | **NF-κB, MAPK** | Inflammatory mediators such as **IL-1β, IL-6**, and **TSLP** ↑  Barrier-related proteins like **filaggrin** and **loricrin** ↓ | GPR15L-GPR15 axis → **T** **cells**.  Recruitment and activation of **DCs, macrophages**.  Cytokines and chemokines influence immune cell responses. |
| Kim et al., 2007 (42) | USA, South Korea | Human keratinocyte cultures and skin biopsies | Skin | Th2 cytokines (**IL-4 and IL-13**) | -- | Macrophage Inflammatory Protein-3 alpha (**MIP-3α)** expression ↓ in AD compared to PS | MIP-3α → **Langerhans cells** to the epidermis.  Chemokines → **neutrophils** and **other immune cells** to sites of infection/inflammation. |
| Komine et al., 1996 (43) | USA | Human keratinocyte cultures and skin biopsies | Skin | -- | **IFN-γ/STAT1, IL-6/STAT1, JAK/TYK,**  **MAPK/Ras** | -- | **Th1 cells** produce IFN-γ (PS).  **Th2 Cells** produce IL-4 and IL-10 (AD).  **Macrophages** and **DCs:** cytokine signaling, immune modulation in inflammatory skin conditions |
| Mascia et al., 2003 (44) | Italy | Human keratinocyte cultures and mouse model | Skin | **TNF-α**, **IFN-γ** | **TNF-α**, **IFN-γ** activates **EGFR** (metalloproteinase-mediated ligand release and ERK phosphorylation) | EGFR activation → **CCL2, CCL5,** and **CXCL10** ↓while **CXCL8** ↑ → modulated inflammation | **CD4+ T cells** and **CD11b+ myeloid cells** are recruited by keratinocyte-derived chemokines during inflammation. |
| Morgenstern et al., 2024 (45) | USA | Human keratinocyte cultures | Skin | **IL-4**, **IL-13 (AD)**  **IL-22 (PS)** | **--** | **IL-4** and **IL-13** → bacterial internalization ↓ and bacterial killing ↑ in undifferentiated keratinocytes.  **IL-22** → bacterial internalization ↑ in differentiated keratinocytes. | **Th2 cells** secrete IL-4, IL-13 while **Th17 cells** secrete IL-22.  Potential downstream interaction with **macrophages** and **neutrophils** involved in inflammation and bacterial clearance |
| Nickoloff et al., 1993 (46) | USA | Human keratinocyte cultures | Skin | **IFN-γ,** bacterial superantigens (e.g., staphylococcal enterotoxins A and B) | -- | **IFN-γ** → **MHC class II**, **ICAM-1** ↑ | ICAM-1 interacts with **LFA-1** on **T cells** → costimulatory signals for superantigen-mediated T cell activation. |
| Semini et al., 2014 (47) | Germany | Human keratinocyte cultures | Skin | **IL-1α, IL-17, IL-22, TNF-α,** and **OSM** | -- | Inflammatory markers, MHC class II, CIITA ↑ | MHC-mediated antigen presentation → **T cell** activation.  Inflammatory cytokines → potential interactions with **DC**s |
| Zhang et al., 2023 (48) | Germany | Skin biopsies and blood samples | Skin | **AD: IL-13,** **IL-22**  **PS:** **IL-17A,** **IL-17F** | -- | **AD:** stromal cells such as fibroblasts and pericytes involved.  **PS:** vascular endothelial cells involved. | **AD:** **Th2**, **Th22 cells** (Type-2 inflammation); interactions with **DC**s and **macrophages**.  **PS:** **Th17**, **Tc17** cells (Type-3 inflammation); interactions with **inflammatory** **myeloid cells** and **circulating immune cells**. |
| Bernard et al., 2012 (49) | France | Human keratinocyte cultures | Skin | **AD: IL-4, IL-13, IL-22**  **PS: IL-17A, IL-22, TNFα** | -- | **AD:** **Type-2 inflammation**→ AMP ↓ and skin barrier dysfunction.  **PS:** **Type-3 inflammation** → keratinocyte proliferation ↑, antimicrobial defense ↑. | **AD:** **Th2**, **Th22 cells** interact with **DCs** to drive inflammation; **macrophages** → chronic lesions. **PS:** **Th17, Th1 cells** interact with keratinocytes and **DCs** to sustain inflammatory cascades; **neutrophils** recruited by chemokines like CXCL8. |

TSLP, Thymic Stromal Lymphopoietin. S. aureus, Staphylococcus aureus. TLR, Toll-like Receptor. EGFR, Epidermal Growth Factor Receptor. JAK, Janus Kinase. DC, Dendritic Cells. SNARE, Soluble N-ethylmaleimide-sensitive factor attachment protein receptors. APC, Antigen-presenting Cell. TNF, Tumor necrosis factor. IFN-γ, interferon-gamma. DAMP, Damage-associated Molecular Patterns. AMP, Anti-Microbial Peptide. **OSM**, Oncostatin M. ICAM-1, Intercellular Adhesion Molecule 1. LFA-1, Lymphocyte Function-associated Antigen 1. CIITA, Master Regulator of MHC gene transcription. “→”:lead to/cause/recruit. “↑”: upregulation/overexpression/increased. “↓”: downregulation/reduced.

1. Al Kindi A, Williams H, Matsuda K, Alkahtani AM, Saville C, Bennett H, et al. Staphylococcus aureus second immunoglobulin-binding protein drives atopic dermatitis via IL-33. J Allergy Clin Immunol. 2021;147(4):1354-68.e3.

2. Ardern-Jones MR, Black AP, Bateman EA, Ogg GS. Bacterial superantigen facilitates epithelial presentation of allergen to T helper 2 cells. Proc Natl Acad Sci U S A. 2007;104(13):5557-62.

3. Aries MF, Hernandez-Pigeon H, Vaissière C, Delga H, Caruana A, Lévêque M, et al. Anti-inflammatory and immunomodulatory effects of Aquaphilus dolomiae extract on in vitro models. Clin Cosmet Investig Dermatol. 2016;9:421-34.

4. Bilsborough J, Leung DY, Maurer M, Howell M, Boguniewicz M, Yao L, et al. IL-31 is associated with cutaneous lymphocyte antigen-positive skin homing T cells in patients with atopic dermatitis. J Allergy Clin Immunol. 2006;117(2):418-25.

5. Buddenkotte J, Stroh C, Engels IH, Moormann C, Shpacovitch VM, Seeliger S, et al. Agonists of proteinase-activated receptor-2 stimulate upregulation of intercellular cell adhesion molecule-1 in primary human keratinocytes via activation of NF-kappa B. J Invest Dermatol. 2005;124(1):38-45.

6. Dai X, Sayama K, Tohyama M, Shirakata Y, Hanakawa Y, Tokumaru S, et al. Mite allergen is a danger signal for the skin via activation of inflammasome in keratinocytes. J Allergy Clin Immunol. 2011;127(3):806-14.e1-4.

7. Dai X, Tohyama M, Murakami M, Shiraishi K, Liu S, Mori H, et al. House dust mite allergens induce interleukin 33 (IL-33) synthesis and release from keratinocytes via ATP-mediated extracellular signaling. Biochim Biophys Acta Mol Basis Dis. 2020;1866(5):165719.

8. Haertlé J, Kienlin P, Begemann G, Werfel T, Roesner LM. Inhibition of IL-17 ameliorates keratinocyte-borne cytokine responses in an in vitro model for house-dust-mite triggered atopic dermatitis. Sci Rep. 2023;13(1):16628.

9. He R, Oyoshi MK, Garibyan L, Kumar L, Ziegler SF, Geha RS. TSLP acts on infiltrating effector T cells to drive allergic skin inflammation. Proc Natl Acad Sci U S A. 2008;105(33):11875-80.

10. Howell MD, Boguniewicz M, Pastore S, Novak N, Bieber T, Girolomoni G, et al. Mechanism of HBD-3 deficiency in atopic dermatitis. Clin Immunol. 2006;121(3):332-8.

11. Howell MD, Fairchild HR, Kim BE, Bin L, Boguniewicz M, Redzic JS, et al. Th2 cytokines act on S100/A11 to downregulate keratinocyte differentiation. J Invest Dermatol. 2008;128(9):2248-58.

12. Hvid M, Vestergaard C, Kemp K, Christensen GB, Deleuran B, Deleuran M. IL-25 in atopic dermatitis: a possible link between inflammation and skin barrier dysfunction? J Invest Dermatol. 2011;131(1):150-7.

13. Ishibashi Y, Sugita T, Nishikawa A. Cytokine secretion profile of human keratinocytes exposed to Malassezia yeasts. FEMS Immunol Med Microbiol. 2006;48(3):400-9.

14. Jang YH, Choi JK, Jin M, Choi YA, Ryoo ZY, Lee HS, et al. House Dust Mite Increases pro-Th2 Cytokines IL-25 and IL-33 via the Activation of TLR1/6 Signaling. J Invest Dermatol. 2017;137(11):2354-61.

15. Jin S, Park CO, Shin JU, Noh JY, Lee YS, Lee NR, et al. DAMP molecules S100A9 and S100A8 activated by IL-17A and house-dust mites are increased in atopic dermatitis. Exp Dermatol. 2014;23(12):938-41.

16. Junghans V, Jung T, Neumann C. Human keratinocytes constitutively express IL-4 receptor molecules and respond to IL-4 with an increase in B7/BB1 expression. Exp Dermatol. 1996;5(6):316-24.

17. Kim MH, Jin SP, Jang S, Choi JY, Chung DH, Lee DH, et al. IL-17A-Producing Innate Lymphoid Cells Promote Skin Inflammation by Inducing IL-33-Driven Type 2 Immune Responses. J Invest Dermatol. 2020;140(4):827-37.e9.

18. Kubo T, Sato S, Hida T, Minowa T, Hirohashi Y, Tsukahara T, et al. IL-13 modulates ∆Np63 levels causing altered expression of barrier- and inflammation-related molecules in human keratinocytes: A possible explanation for chronicity of atopic dermatitis. Immun Inflamm Dis. 2021;9(3):734-45.

19. Kumagai A, Kubo T, Kawata K, Kamekura R, Yamashita K, Jitsukawa S, et al. Keratinocytes in atopic dermatitis express abundant ΔNp73 regulating thymic stromal lymphopoietin production via NF-κB. J Dermatol Sci. 2017;88(2):175-83.

20. Lee H, Ryu WI, Kim HJ, Bae HC, Ryu HJ, Shin JJ, et al. TSLP Down-Regulates S100A7 and ß-Defensin 2 Via the JAK2/STAT3-Dependent Mechanism. J Invest Dermatol. 2016;136(12):2427-35.

21. Lou H, Lu J, Choi EB, Oh MH, Jeong M, Barmettler S, et al. Expression of IL-22 in the Skin Causes Th2-Biased Immunity, Epidermal Barrier Dysfunction, and Pruritus via Stimulating Epithelial Th2 Cytokines and the GRP Pathway. J Immunol. 2017;198(7):2543-55.

22. Lu Z, Xiao S, Chen W, Zhu R, Yang H, Steinhoff M, et al. IL-20 promotes cutaneous inflammation and peripheral itch sensation in atopic dermatitis. Faseb j. 2022;36(6):e22334.

23. Lugović L, Lipozencić J, Jakić-Razumović J. Prominent involvement of activated Th1-subset of T-cells and increased expression of receptor for IFN-gamma on keratinocytes in atopic dermatitis acute skin lesions. Int Arch Allergy Immunol. 2005;137(2):125-33.

24. Luo CH, Lai AC, Tsai CC, Chen WY, Chang YS, Chung EJ, et al. Staphylococcus aureus exacerbates dermal IL-33/ILC2 axis activation through evoking RIPK3/MLKL-mediated necroptosis of dry skin. JCI Insight. 2024;9(6).

25. Meng J, Wang J, Buddenkotte J, Buhl T, Steinhoff M. Role of SNAREs in Atopic Dermatitis-Related Cytokine Secretion and Skin-Nerve Communication. J Invest Dermatol. 2019;139(11):2324-33.

26. Moriwaki M, Iwamoto K, Niitsu Y, Matsushima A, Yanase Y, Hisatsune J, et al. Staphylococcus aureus from atopic dermatitis skin accumulates in the lysosomes of keratinocytes with induction of IL-1α secretion via TLR9. Allergy. 2019;74(3):560-71.

27. Murthy A, Shao YW, Narala SR, Molyneux SD, Zúñiga-Pflücker JC, Khokha R. Notch activation by the metalloproteinase ADAM17 regulates myeloproliferation and atopic barrier immunity by suppressing epithelial cytokine synthesis. Immunity. 2012;36(1):105-19.

28. Ogawa T, Ishitsuka Y, Nakamura Y, Kubota N, Saito A, Fujisawa Y, et al. NRF2 Augments Epidermal Antioxidant Defenses and Promotes Atopy. J Immunol. 2020;205(4):907-14.

29. Park HR, Oh JH, Lee YJ, Park SH, Lee YW, Lee S, et al. Inflammasome-mediated Inflammation by Malassezia in human keratinocytes: A comparative analysis with different strains. Mycoses. 2021;64(3):292-9.

30. Proper SP, Dwyer AT, Appiagyei A, Felton JM, Ben-Baruch Morgenstern N, Marlman JM, et al. Aryl hydrocarbon receptor and IL-13 signaling crosstalk in human keratinocytes and atopic dermatitis. Front Allergy. 2024;5:1323405.

31. Qian X, Tong M, Zhang T, Li Q, Hua M, Zhou N, et al. IL-24 promotes atopic dermatitis-like inflammation through driving MRSA-induced allergic responses. Protein Cell. 2024.

32. Salimi M, Xue L, Jolin H, Hardman C, Cousins DJ, McKenzie AN, et al. Group 2 Innate Lymphoid Cells Express Functional NKp30 Receptor Inducing Type 2 Cytokine Production. J Immunol. 2016;196(1):45-54.

33. Savinko T, Matikainen S, Saarialho-Kere U, Lehto M, Wang G, Lehtimäki S, et al. IL-33 and ST2 in atopic dermatitis: expression profiles and modulation by triggering factors. J Invest Dermatol. 2012;132(5):1392-400.

34. Sawada Y, Honda T, Nakamizo S, Nakajima S, Nonomura Y, Otsuka A, et al. Prostaglandin E(2) (PGE(2))-EP2 signaling negatively regulates murine atopic dermatitis-like skin inflammation by suppressing thymic stromal lymphopoietin expression. J Allergy Clin Immunol. 2019;144(5):1265-73.e9.

35. Su H, Luo Y, Sun J, Liu X, Ling S, Xu B, et al. Transglutaminase 3 Promotes Skin Inflammation in Atopic Dermatitis by Activating Monocyte-Derived Dendritic Cells via DC-SIGN. J Invest Dermatol. 2020;140(2):370-9.e8.

36. Suárez-Fariñas M, Dhingra N, Gittler J, Shemer A, Cardinale I, de Guzman Strong C, et al. Intrinsic atopic dermatitis shows similar TH2 and higher TH17 immune activation compared with extrinsic atopic dermatitis. J Allergy Clin Immunol. 2013;132(2):361-70.

37. Takai T, Chen X, Xie Y, Vu AT, Le TA, Kinoshita H, et al. TSLP expression induced via Toll-like receptor pathways in human keratinocytes. Methods Enzymol. 2014;535:371-87.

38. Yoshikawa Y, Sasahara Y, Takeuchi K, Tsujimoto Y, Hashida-Okado T, Kitano Y, et al. Transcriptional Analysis of Hair Follicle-Derived Keratinocytes from Donors with Atopic Dermatitis Reveals Enhanced Induction of IL32 Gene by IFN-γ. Int J Mol Sci. 2013;14(2):3215-27.

39. Boniface K, Diveu C, Morel F, Pedretti N, Froger J, Ravon E, et al. Oncostatin M secreted by skin infiltrating T lymphocytes is a potent keratinocyte activator involved in skin inflammation. J Immunol. 2007;178(7):4615-22.

40. Corrêa MP, Correia-Silva RD, Sasso GRS, D'Ávila S CGP, Greco KV, Oliani SM, et al. Expression Pattern and Immunoregulatory Roles of Galectin-1 and Galectin-3 in Atopic Dermatitis and Psoriasis. Inflammation. 2022;45(3):1133-45.

41. Dainichi T, Nakano Y, Doi H, Nakamizo S, Nakajima S, Matsumoto R, et al. C10orf99/GPR15L Regulates Proinflammatory Response of Keratinocytes and Barrier Formation of the Skin. Front Immunol. 2022;13:825032.

42. Kim BE, Leung DY, Streib JE, Kisich K, Boguniewicz M, Hamid QA, et al. Macrophage inflammatory protein 3alpha deficiency in atopic dermatitis skin and role in innate immune response to vaccinia virus. J Allergy Clin Immunol. 2007;119(2):457-63.

43. Komine M, Freedberg IM, Blumenberg M. Regulation of epidermal expression of keratin K17 in inflammatory skin diseases. J Invest Dermatol. 1996;107(4):569-75.

44. Mascia F, Mariani V, Girolomoni G, Pastore S. Blockade of the EGF receptor induces a deranged chemokine expression in keratinocytes leading to enhanced skin inflammation. Am J Pathol. 2003;163(1):303-12.

45. Morgenstern AR, Peterson LF, Arnold KA, Brewer MG. Differentiation of keratinocytes or exposure to type 2 cytokines diminishes S. aureus internalization. mSphere. 2024;9(4):e0068523.

46. Nickoloff BJ, Mitra RS, Green J, Zheng XG, Shimizu Y, Thompson C, et al. Accessory cell function of keratinocytes for superantigens. Dependence on lymphocyte function-associated antigen-1/intercellular adhesion molecule-1 interaction. J Immunol. 1993;150(6):2148-59.

47. Semini G, Hildmann A, Klein A, Lucka L, Schön M, Schön MP, et al. Inositol-C2-PAF down-regulates components of the antigen presentation machinery in a 2D-model of epidermal inflammation. Biochem Pharmacol. 2014;87(3):477-88.

48. Zhang B, Roesner LM, Traidl S, Koeken V, Xu CJ, Werfel T, et al. Single-cell profiles reveal distinctive immune response in atopic dermatitis in contrast to psoriasis. Allergy. 2023;78(2):439-53.

49. Bernard FX, Morel F, Camus M, Pedretti N, Barrault C, Garnier J, et al. Keratinocytes under Fire of Proinflammatory Cytokines: Bona Fide Innate Immune Cells Involved in the Physiopathology of Chronic Atopic Dermatitis and Psoriasis. J Allergy (Cairo). 2012;2012:718725.
