# Supplementary material for: Keratinocytes as active regulators of cutaneous and mucosal immunity: a systematic review across inflammatory epithelial disorders
Source: Front Immunol. 2025 Dec 17;16:1694066. doi: 10.3389/fimmu.2025.1694066 (PMC12753988; doi:10.3389/fimmu.2025.1694066)
Supplement: Supplementary file 1 [file DataSheet1.zip › Supplementary Table 3.DOCX]

**Table S3 Summary of Studies Investigating the Role of Keratinocytes in Lichen Planus**

| **Author & Year** | **Country** | **Model** | **Tissue** | **Trigger** | **Key Pathways in Keratinocytes** | **Keratinocyte Response** | **Interaction with Immune Cells** |
| --- | --- | --- | --- | --- | --- | --- | --- |
| Adami et al., 2014 (1) | USA | Oral mucosal biopsies from patients with OLP | Mucosa | PAMPs | **TLRs,** particularly TLR1 → **NF-κB** | Production of chemokines like **CXCL1** and **IL8** upon **TLR** activation | **T cells and neutrophils recruited** through CXCL1 and IL8 into subepithelial space. |
| Bramanti et al.,1995 (2) | USA | Oral mucosal biopsies from patients with OLP and healthy controls | Mucosa | -- | -- | **HSPs**↑ in basal keratinocytes | **γδ T cells** increased absolute numbers in OLP lesions but represented < 1% **CD3+ T cells** |
| Danielsson et al., 2013 (3) | Sweden, UK | Oral mucosal biopsies from patients with OLP | Mucosa | **--** | -- | In whole tissue sample: **CXCL10, CXCL11**, and **CXCR3** ↑**;**  **ELF-3** transcription factor ↓ → impaired keratinocyte differentiation | **T cells** express CXCR3 in response to chemokine signaling → **Th1 immune response** and chronic inflammation. |
| Khan et al., 2003 (4) | Australia | Oral mucosal biopsies from patients with OLP | Mucosa | **--** | -- | **TNF-α** is secreted by basal keratinocytes and interacts with its receptor (TNF R1) on keratinocytes and infiltrating T cells.  **TGF-β1** is weakly expressed by keratinocytes in OLP, with its immunosuppressive potential possibly blocked by IFN-γ in OLP lesions. | **TNF-α** and **IFN-γ** secreted by infiltrating mononuclear cells promote **Th1-dominant** immune response.  Intraepithelial **CD8+ cytotoxic T cells** trigger keratinocyte apoptosis.  **CD4+ regulatory T cells** regulate immunity in the lamina propria.  **Langerhans cells** may assist in antigen presentation alongside keratinocytes. |
| Lauffer et al., 2018 (5) | Germany | Skin biopsies from patients with lichen planus (LP) and lupus erythematosus (LE), alongside keratinocyte cultures | Skin | IFN-γ and TNF-α | RIP3 and pMLKL | Apoptosis and necroptosis | Dominant Th1/Tc1 type **CD4+ and CD8+ T cells** produce IFN-γ and TNF-α.  **pDCs** drive type I interferon responses, amplifying T-cell-mediated inflammation. |
| Ma et al., 2019 (6) | China | Human oral keratinocytes and Jurkat T cells co-culture system, with tissue samples from OLP patients | Mucosa | **IGF1;**  cytokine networks (e.g., **IFN-γ** and **IL-4**) | Cytokine networks → PI3K/AKT/MTOR | **IGF1** → keratinocyte proliferation and T cell apoptosis | **CD4+ T cells i**nteract with keratinocytes via cytokine networks, which influence apoptosis and proliferation. |
| Santoro et al., 2003 (7) | Italy | Human biopsies of oral lichen planus OLP and CLP patients | Both skin and mucosa | -- | **NF-κB** (more pronounced in OLP than in CLP, aligned with OLP’s chronic nature) | **TNF-α, IL-1β**, adhesion molecules like **ICAM-1** ↑ | Recruitment and activation of cytotoxic **CD8+ T cells,** which contribute to keratinocyte damage, and **CD4+ T cells** which contribute to inflammatory cytokine production. |
| Takeuchi et al., 1988 (8) | Japan | Oral mucosal biopsies from patients with OLP | Mucosa | **IFN-γ** | -- | **MHC class I** and II expression ↑ | **CD8+ T cells** damage keratinocytes, predominant in areas with satellite cell necrosis (SCN) where **CD4+ T cells** are less dominant.  **Monocyte/macrophage** subsets and vascular endothelial cells presents antigen. |
| Tan et al., 2023 (9) | China | Human keratinocyte cultures | Mucosa | -- | **IGF1 Pathway** | IGF1 reverses apoptosis in keratinocytes co-cultured with T cells showing abnormal ATG9B expression | Dysregulated autophagy mediated by **ATG9B** in T cells → cytokine (e.g., IFN-γ, TNF-α) secretion ↑ → keratinocyte apoptosis and **Th1-dominant** immune response in OLP lesions |
| Zhang et al., 2017 (10) | China | Human keratinocyte cultures | Mucosa | LPS | **TLR4, NF-κB,** and **PI3K/mTOR** | **B7-H1 (PD-L1)** ↑ | T-cell proliferation ↓ and apoptosis ↑ → immune tolerance in OLP |

OLP, Oral Lichen Planus. CLP, Cutaneous Lichen Planus. TLR, Toll-like Receptor. HSP, Heat Shock Protein. pDC, Plasmacytoid Dendritic Cell. IGF1, Insulin-like Growth Factor 1. ELF-3, E74-like ETS transcription factor 3. TNF-α, Tumor Necorsis Factor-alpha. ICAM-1 , Intracellular Adhesion Molecule-1. ATG9B, Autophagy-related protein 9B. →: lead to/causes. ↑: upregulation/overexpression/increased. ↓: downregulation/decreased

1. Adami GR, Yeung AC, Stucki G, Kolokythas A, Sroussi HY, Cabay RJ, et al. Gene expression based evidence of innate immune response activation in the epithelium with oral lichen planus. Arch Oral Biol. 2014;59(3):354-61.

2. Bramanti TE, Dekker NP, Lozada-Nur F, Sauk JJ, Regezi JA. Heat shock (stress) proteins and gamma delta T lymphocytes in oral lichen planus. Oral Surg Oral Med Oral Pathol Oral Radiol Endod. 1995;80(6):698-704.

3. Danielsson K, Boldrup L, Rentoft M, Coates PJ, Ebrahimi M, Nylander E, et al. Autoantibodies and decreased expression of the transcription factor ELF-3 together with increased chemokine pathways support an autoimmune phenotype and altered differentiation in lichen planus located in oral mucosa. J Eur Acad Dermatol Venereol. 2013;27(11):1410-6.

4. Khan A, Farah CS, Savage NW, Walsh LJ, Harbrow DJ, Sugerman PB. Th1 cytokines in oral lichen planus. J Oral Pathol Med. 2003;32(2):77-83.

5. Lauffer F, Jargosch M, Krause L, Garzorz-Stark N, Franz R, Roenneberg S, et al. Type I Immune Response Induces Keratinocyte Necroptosis and Is Associated with Interface Dermatitis. J Invest Dermatol. 2018;138(8):1785-94.

6. Ma RJ, Tan YQ, Zhou G. Aberrant IGF1-PI3K/AKT/MTOR signaling pathway regulates the local immunity of oral lichen planus. Immunobiology. 2019;224(3):455-61.

7. Santoro A, Majorana A, Bardellini E, Festa S, Sapelli P, Facchetti F. NF-kappaB expression in oral and cutaneous lichen planus. J Pathol. 2003;201(3):466-72.

8. Takeuchi Y, Tohnai I, Kaneda T, Nagura H. Immunohistochemical analysis of cells in mucosal lesions of oral lichen planus. J Oral Pathol. 1988;17(8):367-73.

9. Tan YQ, Zhang J, Zhou G. Autophagy-related 9 homolog B regulates T-cell-mediated immune responses in oral lichen planus. Arch Oral Biol. 2023;146:105589.

10. Zhang J, Tan YQ, Wei MH, Ye XJ, Chen GY, Lu R, et al. TLR4-induced B7-H1 on keratinocytes negatively regulates CD4(+) T cells and CD8(+) T cells responses in oral lichen planus. Exp Dermatol. 2017;26(5):409-15.
