# Supplementary material for: Keratinocytes as active regulators of cutaneous and mucosal immunity: a systematic review across inflammatory epithelial disorders
Source: Front Immunol. 2025 Dec 17;16:1694066. doi: 10.3389/fimmu.2025.1694066 (PMC12753988; doi:10.3389/fimmu.2025.1694066)
Supplement: Supplementary file 1 [file DataSheet1.zip › Supplementary Table 4.DOCX]

**Table S4** Summary of Studies Investigating the Role of Keratinocytes in Bullous Pemphigoid, and Systemic as well as Cutaneous Lupus Erythematosus

| **Author & Year** | **Country** | **Model** | **Skin Condition** | **Tissue** | **Trigger** | **Key Pathways in Keratinocytes** | **Keratinocyte Response** | **Interaction with Immune Cells** |
| --- | --- | --- | --- | --- | --- | --- | --- | --- |
| Frezzolini et al., 2004 (1) | Italy | Human skin biopsies | Bullous Pemphigoid | Skin | **--** | -- | **IL-16,** chemotaxins like **eotaxin** and **TARC** production↑ | IL-16 recruits **CD4+ T cells** at lesional sites.  Keratinocyte-derived chemokines recruits **eosinophils** and **Th2 cells** → blister formation and tissue damage |
| Zhang et al., 2018 (2) | USA, China | Mouse model and human skin biopsies | Bullous Pemphigoid | Skin | **TNF-α**, BP180 dysfunction | -- | **TSLP**↑ → itch and inflammation independent of histamine and adaptive immunity | **Neutrophils, eosinophils,** and **mast cells** ↑ infiltration into dermis, but no T or B cells needed in initiating inflammation. |
| Lampert et al, 1984 (3) | United Kingdom | Human skin biopsies | Various inflammatory dermatoses, including Bullous Pemphigoid, Lichen Planus, discoid Lupus erythematosus, and Pemphigus Vulgaris | Skin | Lymphoid infiltration, likely mediated by interferons | -- | Induction of **MHC class II (HLA-DR)** antigen presentation | **Lymphoid cells** infiltrate and cause immune-mediated damage.  **Langerhans cells** show increased presence and dendritic activity in affected areas. |
| Psarras et al., 2020 (4) | United Kingdom | Human skin biopsies | Systemic Lupus Erythematosus | Skin | **IFN-κ** expression enhanced by environmental triggers like **UV radiation** | -- | Primary producers of type I interferons (**IFN-κ and IFN-α**) | Keratinocytes primarily contribute to the local immune environment by producing type I IFNs independent of infiltrating immune cells |
| Fetter et al., 2020 (5) | Germany | Human skin biopsies, keratinocyte cultures and Mouse Model | Cutaneous Lupus Erythematosus | Skin | **DAMPs.**  **Type I interferons (IFNs)** | **DAMPs**→TLR-dependent and -independent pathways.  **Type I IFNs** →**JAK/STAT** | **IFNs** → pro-inflammatory chemokines and mediators↑ (**CXCL9, CXCL10, CXCL11** and **TRAIL, AIM2,** and **BLyS).**  DAMPs → IFNs↑ → autocrine IFN signaling → inflammation and keratinocyte necroptosis. | Chemokines recruits **CXCR3+ cytotoxic T** **cells** and **pDC**s into lesional skin→ keratinocyte necroptosis and inflammation |
| Lauffer et al., 2018 (6) | Germany | Skin biopsies from patients with lichen planus and lupus erythematosus; keratinocyte cultures | Lichen Planus and Lupus Erythematosus | Skin | IFN-γ and TNF-α | RIP3 and pMLKL | Apoptosis and necroptosis | Dominant Th1/Tc1 type **CD4+ and CD8+ T cells** produce IFN-γ and TNF-α.  **pDCs** drive type I interferon responses, amplifying T-cell-mediated inflammation. |
| Meller et al., 2005 (7) | Germany | Human keratinocytes cultures and skin biopsies from patients with cutaneous lupus erythematosus (CLE) | Cutaneous Lupus Erythematosus | Skin | **IFN-α, IL-1.**  **UV** radiation | -- | **IFN-α** → **CXCR3 ligands**↑ (e.g., **CXCL9, CXCL10**).  UV → **CCL27**↑ released into dermis in early lesions.  UV → inflammatory cytokines↑ **(CCL5, CCL20, CCL22, CXCL8)** in keratinocytes stimulated by IFNa/IL-1. | Activated **pDCs** produce IFN-α.  Synergy between **CXCR3** ligands and homeostatic chemokines (e.g., **CXCL12**) enhances homing of CLA+ memory T cells to skin lesions. |
| Zahn et al., 2011 (8) | Germany | Skin biopsies and human keratinocyte cultures | Cutaneous Lupus Erythematosus, including chronic discoid CLE (CDLE) and subacute CLE (SCLE) | Skin | Immunostimulatory nucleic acids.  **Type III interferon** (IFNλ) | **TLR** 3 stimulated by immunostimulatory nucleic acids;  **JAK1/STAT** by IFNλ | **TLR3 stimulation**→ IFNλ release→ autocrine induction of pro-inflammatory cytokines such as **CXCL9** and **CCL3** | CXCL9 recruits **pDC**s (produce **IFN-α)** and **cytotoxic T cells** (produce IFNγ) at dermo-epidermal junction → **CXCL10** expression in keratinocytes → interface dernatitis |

TSLP, Thymic Stromal Lymphopoietin. DAMP, Danger-Associated Molecular Pattern. pDC, Plasmacytoid Dendritic Cell. TLR, Toll-like receptor. RIP3, Receptor-Interacting-Protein-Kinase 3. pMLKL, Phosphorylated Mixed Lineage Kinase Domain Like Pseudokinase. CLA, Common Leukocyte Antigen.

1. Frezzolini A, Cianchini G, Ruffelli M, Cadoni S, Puddu P, De Pità O. Interleukin-16 expression and release in bullous pemphigoid. Clin Exp Immunol. 2004;137(3):595-600.

2. Zhang Y, Hwang BJ, Liu Z, Li N, Lough K, Williams SE, et al. BP180 dysfunction triggers spontaneous skin inflammation in mice. Proc Natl Acad Sci U S A. 2018;115(25):6434-9.

3. Lampert IA. Expression of HLA-DR (Ia like) antigen on epidermal keratinocytes in human dermatoses. Clin Exp Immunol. 1984;57(1):93-100.

4. Psarras A, Alase A, Antanaviciute A, Carr IM, Md Yusof MY, Wittmann M, et al. Functionally impaired plasmacytoid dendritic cells and non-haematopoietic sources of type I interferon characterize human autoimmunity. Nat Commun. 2020;11(1):6149.

5. Fetter T, Smith P, Guel T, Braegelmann C, Bieber T, Wenzel J. Selective Janus Kinase 1 Inhibition Is a Promising Therapeutic Approach for Lupus Erythematosus Skin Lesions. Front Immunol. 2020;11:344.

6. Lauffer F, Jargosch M, Krause L, Garzorz-Stark N, Franz R, Roenneberg S, et al. Type I Immune Response Induces Keratinocyte Necroptosis and Is Associated with Interface Dermatitis. J Invest Dermatol. 2018;138(8):1785-94.

7. Meller S, Winterberg F, Gilliet M, Müller A, Lauceviciute I, Rieker J, et al. Ultraviolet radiation-induced injury, chemokines, and leukocyte recruitment: An amplification cycle triggering cutaneous lupus erythematosus. Arthritis Rheum. 2005;52(5):1504-16.

8. Zahn S, Rehkämper C, Kümmerer BM, Ferring-Schmidt S, Bieber T, Tüting T, et al. Evidence for a pathophysiological role of keratinocyte-derived type III interferon (IFNλ) in cutaneous lupus erythematosus. J Invest Dermatol. 2011;131(1):133-40.

9. Tam C, Mun JJ, Evans DJ, Fleiszig SM. Cytokeratins mediate epithelial innate defense through their antimicrobial properties. J Clin Invest. 2012;122(10):3665-77.
