# Supplementary material for: Keratinocytes as active regulators of cutaneous and mucosal immunity: a systematic review across inflammatory epithelial disorders
Source: Front Immunol. 2025 Dec 17;16:1694066. doi: 10.3389/fimmu.2025.1694066 (PMC12753988; doi:10.3389/fimmu.2025.1694066)
Supplement: Supplementary file 1 [file DataSheet1.zip › Supplementary Table 5.DOCX]

**Table S5** Summary of Studies Investigating the Role of Keratinocytes in Graft-versus-Host Disease (GvHD)

| **Author & Year** | **Country** | **Model** | **Tissue** | **Trigger** | **Key Pathways in Keratinocytes** | **Keratinocyte Response** | **Interaction with Immune Cells** |
| --- | --- | --- | --- | --- | --- | --- | --- |
| Brüggen et al., 2014 (1) | Austria | Human lesional skin biopsies | Skin | **Acute GvHD**: TNF-α, IL-1α, Th2 cytokines (IL-4, IL-5, IL-13, and chemokine CCL17) in response to conditioning prior to HCT  **Chronic lichenoid GvHD:** IFN-γ and IL-17 producing CD8+ T cells  **Chronic sclerotic GvHD**: -- | **Acute GvHD:** --  **Chronic lichenoid GvHD:** Granzyme/perforin, Fas/FasL  **Chronic sclerotic GvHD**: TRAIL receptor signaling | **Acute GvHD:** TSLP ↑  **Chronic lichenoid GvHD:** apoptosis  **Chronic sclerotic GvHD**: | **Acute GvHD:** T cell recruitment, Th2 responses (IL-4, IL-22, CCL17)  **Chronic lichenoid GvHD:** Recruitment of CD8+ T cells by CXCR3 ligands (CXCL9, CXCL10) and CCR5 ligands (CCL5) on keratinocytes  **Chronic sclerotic GvHD**: Mast cells and Th1 CD8+ T cells interacting with keratinocytes through TRAIL |
| Imanguli et al., 2009 (2) | USA | Oral mucosal biopsies | Mucosa | **Type I IFN** produced by **pDCs** | **IFN** signaling pathway | CXCL9, IL-15, Type I IFN-inducible factors (e.g., MxA) ↑  Apoptosis | **CD4+ T cells** present in later stages as immune regulation.  **Macrophages** infiltrate tissues and produce IL-15  T-bet+ and granzyme B+ **CD8+ T cells** triggers keratinocyte apoptosis.  Type I IFN produced by **pDCs** amplifies T cell responses and keratinocyte apoptosis |
| Kim et al., 2009 (3) | USA, Netherlands | Transgenic mouse model | Skin | IFN-γ | -- | **MHC class I** and **II** ↑ | In the absence of **LCs** and **dDCs,** keratinocytes act as accessory cells to present endogenous antigens to prime naïve **CD8+ T cells** (OT-I cells) |
| Strobl et al., 2021 (4) | Austria | Human blood samples | Skin | **IL-13** and **IL-17** secreted by cTRMs | -- | Pro-inflammatory cytokines like **TSLP** ↑  Activation and damage | **cTRMs** produce pro-inflammatory **Th2/Th17** cytokines like IL-13 and IL-17 |

TNF, Tumor Necrosis Factor. HCT, Hematopoietic Cell Transplant. TSLP, Thymic stromal lymphopoietin. TRAIL, Tumor Necrosis Factor-related Apoptosis-inducing Ligand. IFN, Type I interferon. pDC, Plasmacytoid Dendritic Cell. LC, Langerhans cell. dDC, dermal dendritic cell. cTRM, Circulating tissue-resident memory T cell. ↑: increased expression or upregulation.

1. Brüggen MC, Klein I, Greinix H, Bauer W, Kuzmina Z, Rabitsch W, et al. Diverse T-cell responses characterize the different manifestations of cutaneous graft-versus-host disease. Blood. 2014;123(2):290-9.

2. Imanguli MM, Swaim WD, League SC, Gress RE, Pavletic SZ, Hakim FT. Increased T-bet+ cytotoxic effectors and type I interferon-mediated processes in chronic graft-versus-host disease of the oral mucosa. Blood. 2009;113(15):3620-30.

3. Kim BS, Miyagawa F, Cho YH, Bennett CL, Clausen BE, Katz SI. Keratinocytes function as accessory cells for presentation of endogenous antigen expressed in the epidermis. J Invest Dermatol. 2009;129(12):2805-17.

4. Strobl J, Gail LM, Kleissl L, Pandey RV, Smejkal V, Huber J, et al. Human resident memory T cells exit the skin and mediate systemic Th2-driven inflammation. J Exp Med. 2021;218(11).
